# New Development in the Preparation of Micro/Nano-Wires of Molecular (Magnetic) Conductors

**DOI:** 10.3390/ma3031640

**Published:** 2010-03-08

**Authors:** Toyonari Sugimoto, Hisashi Tanaka, Dominique de Caro, Lydie Valade

**Affiliations:** 1 Department of Chemistry, Graduate School of Science, Osaka Prefecture University, Osaka 599-8570, Japan; 2 Nanotechnology Research Institute, AIST, Tsukuba 305-8565, Japan; E-Mail: hisashi.tanaka@aist.go.jp (H.T.); 3 CNRS, LCC (Laboratoire de Chimie de Coordination), F-31077 Toulouse, France; E-Mails: decaro@lcc-toulouse.fr (D.C.); lydie.valade@lcc-toulouse.fr (L.V.); 4 Université de Toulouse, UPS, INPT, LCC, F-31077 Toulouse, France

**Keywords:** nano-size molecular transistor/spin-transitor, micro/nano-size wire, molecular (magnetic) conductor, template-free and -assisted methods, size effect on electrical conductivity

## Abstract

A lot of molecular (magnetic) conductors are prepared largely using charge-transfer (CT) salts of donor molecules with acceptor molecules or nonmagnetic or magnetic anions such as metal halides and oxides; their CT salts are usually obtained as bulk crystals, which are used to elucidate the electrical conducting (magnetic) properties. In contrast, a small number of micro/nano-crystals of the molecular (magnetic) conductors, especially micro/nano-wires, are known, of which highly conducting nanowires are necessary as a key component in the development of the next generation of nano-size transistors and spin-transistors. Very recently, we succeeded in preparing highly conductive micro/nano-wires of CT salts between bent donor molecules developed by one of the author’s group and magnetic FeX_4_^–^ (X = Cl, Br) ions: (1) by electrochemical oxidation of the bent donor molecules with a silicon wafer electrode coated with a phospholipid multi-lamellar structure as well as, (ii) by electrochemical oxidation of the bent donor molecules with a large arc structure, in the presence of NBu_4_FeX_4_ supporting electrolytes. This article reviews template-free and template-assisted methods developed so far for the preparation of micro/nano-wires of molecular (magnetic) conductors along with our new methods. The conducting properties of these micro/nano-wires are compared with those of the corresponding bulk crystals.

## 1. Introduction

**Figure 1:**
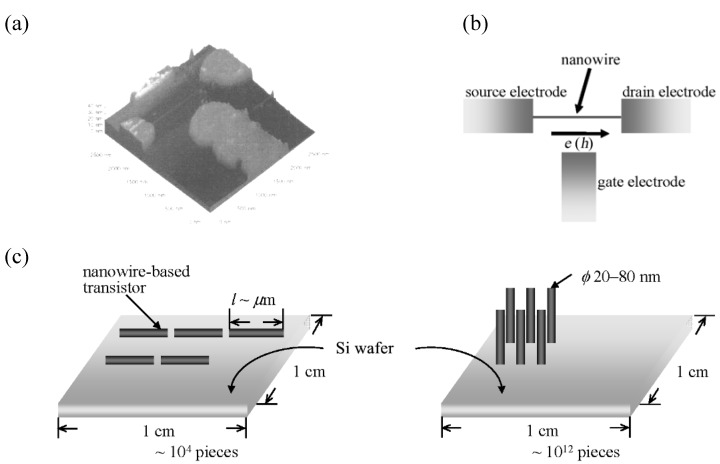
(a) A SEM image of a nano-size transistor fabricated on silicon wafer (b) a schematic diagram, and (c) arrangement of nanowires parallel and perpendicular to a silicon wafer substrate with an area of 1 × 1 cm^2^.

A nanowire is defined as an anisotropic nano-crystal with a large length/diameter ratio, and are expected to play important roles as both interconnectors and functional components in the fabrication of nano-size electronic and optoelectronic devices [1]. In fact, during the past decade a lot of applications of nanowires have been demonstrated such as in lasers, transistors, photodetectors, sensors and energy conversion devices for photovoltaics and thermoelectric [2,3,4]. For a nanowire transistor, a single nanowire with semiconductive property (if the nanowire is metallic, a quantum dot must be introduced near the center of the nanowire) is bridged between source and drain electrodes fabricated on silicon wafer substrate, and a third gate electrode is placed along the direction perpendicular to the nanowire (Figure 1(a)). The flow of electrons or holes between the source and drain electrodes is tuned by changing the voltage of the gate electrode, as shown by a schematic diagram in Figure 1(b). In the case of a nano-size transistor, a very small number of electrons or holes are allowed to flow through the nanowire, so the flow rate becomes remarkably fast compared to that in conventional transistors. This is because the interference between electrons or holes is significantly reduced, giving rise to high-speed and a large amount of information processing. Such a single nanowire-based transistor has at largest a size of several micrometers in length and 20–80 nm in diameter. About 10^4^ pieces of the transistor can be loaded on a silicon wafer substrate with an area of 1 × 1 cm^2^ in the parallel arrangement, while the number of the pieces loaded increases by around 10^3^–times in the perpendicular arrangement (Figure 1(c)).

Metal and semiconductor nanowires based on metal elements, metal oxides and metal chalcogenides, with highly controlled dimensions, orientations and compositions, can now be prepared readily using various solution and gas-phase synthetic methodologies [5]. In contrast, there is still slow progress in the preparation of nanowires based on organic molecules [6]. So far, single crystals of organic molecules require a size as large as possible and a high quality in order to elucidate their structures and physical properties. For this end, a lot of methods have been developed and still much effort is continuing in the search for more efficient methods. On the other hand, it has been very difficult to prepare nano-sized single crystals. However, several efficient methods have recently been developed for the preparation of especially molecular conductors and magnetic conductors, which are expected to be used as conducting wires bridging between two electrodes in nano-size molecular transistors and spin-transistors. This article reviews efficient preparation methods of micro/nano-wires of molecular (magnetic) conductors, some of which have very recently been developed by the authors’ groups.

## 2. Preparation Methods of Micro/Nano-Wires of Molecular (Magnetic) Conductors

To prepare micro/nano-wires of molecular (magnetic) conductors, two methods are so far developed; one which uses a template and one that does not in the mixing between donor and acceptor molecules or in the electrochemical oxidation of donor molecules. The former method includes mixing of highly-dilute donor and acceptor solutions, deposition of donor and acceptor molecules by a dip-coating onto stainless steel, and electrochemical deposition of donor molecules onto conventional native silicon wafer and platinum rod or nano-size electrodes. The latter method uses nano-size channels of supramolecular network composed of counter halide anions and iodine-containing neutral molecules, and of porous alumina and phospholipid multi-lamellar membranes coated on gold, silver or silicon wafer in the electrochemical oxidation of donor molecules.

### 2.1. Template-Free Method

#### 2.1.1. Two Liquid Phase Mixing

**Figure 2:**
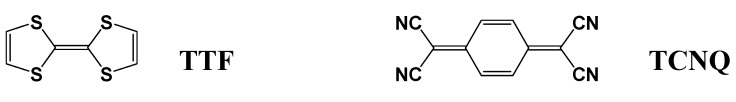
The chemical structures of tetrathiafulvalene (**TTF**) and tetracyanoquino-dimethane (**TCNQ**).

**Figure 3:**
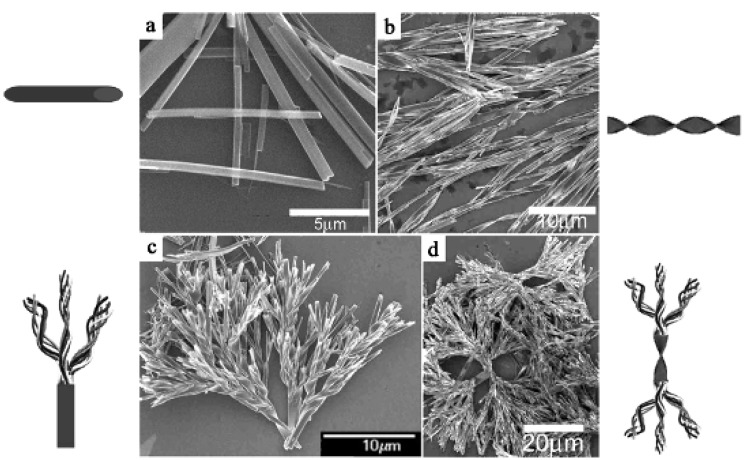
SEM images of **TTF**•**TCNQ** complex morphologies as prepared at the drip rate of (a) 1 μL s^−1^, **TTF**•**TCNQ** nanowires; (b) 0.02 mL s^−1^, **TTF**•**TCNQ** helical nanowires; (c) 0.04 mL s^−1^, **TTF**•**TCNQ** helical dendrites; (d) 0.5 mL s^−1^, **TTF**•**TCN** complicated helical dendrites. [Reproduced with permission from [7] *Nanotechnology* **2007**, *18*, 495704:1–495704:7. ©2007, IOP Publishing Ltd].

A tetracyanoquinodimethane (**TCNQ)** (Figure 2) solution in hexane (0.03 M, 10 mL) is dripped into a tetrathiafulvalene (**TTF)** (Figure 2) solution in acetonitrile (0.025 M, 10 mL) with stirring at different rates of 1, 20, 40, and 500 μL s^–1^, and at different temperatures of –50, –10, 0, and 50 °C [7]. The drip rate of 1 μL s^–1^ gives **TTF**•**TCNQ** nanowires with a diameter of 200–700 nm and length of above 10 μm. Increasing the drip rate to 20 μL s^–1^ facilitates the quenching rate of nucleation, growth and assembly, resulting in the formation of **TTF**•**TCNQ** helical nanowires with a diameter of 200–900 nm and length of several tens of micrometers. Some typical helical dendrites, in which a straight and stiff microwire is directly connected to the starting point of the helical dendrite, are obtained at the drip rate of 40 μL s^–1^. The complicated **TTF**•**TCNQ** helical dendrite is formed at the drip rate of 500 μL s^–1^ and is composed of two helical dendrites connected with each other. Each of the helical dendrites is an assembly of many helical nanowires. Figure 3(a)–(d) show SEM images of **TTF**•**TCNQ** complex morphologies, nanowires, helical nanowires, helical dendrites and complicated helical dendrites as prepared at the drip rates of 1, 20, 40 and 500 μL s^–1^, respectively. The FT–IR spectra of the **TTF**•**TCNQ** complex morphologies also provide evidence about the complex formation between **TTF** and **TCNQ** molecules. All of the **TTF**•**TCNQ** complex morphologies are single crystals, as evidenced by the indexing of the spots in their SAED patterns. In addition to the solution concentration in the reaction, the temperature is also the other key factor influencing the nucleation and growth. At low temperatures, the absolute growth rate of the **TTF**•**TCNQ** complex is low. The π–π stacking interactions between **TTF** molecules and between **TCNQ** molecules quickly lead to the formation of **TTF**•**TCNQ** nanowires. With increasing reaction temperature, the increase absolute growth rates of **TTF**•**TCNQ** complexes may result in a significant strain on the surface of the nanowires, which is likely to result in the transformation of straight nanowires into helical nanowires, eventually evolving into complex growth patterns. As expected, the four kinds of **TTF**•**TCNQ** complex morphologies as described above are obtained at different reaction temperatures. The reaction at –50 °C gives the **TTF**•**TCNQ** nanowires with the diameter of 100–500 nm and the length of a few micrometers to tens of micrometers. As the temperature increases from –50 °C to –10 °C, 0 °C, and 50 °C, the helical wires (diameter = 300–800 nm and length = 3–18 μm), helical dendrites, and the complicated helical dendrites are formed respectively, all of which have high crystallinity.

**Figure 4:**
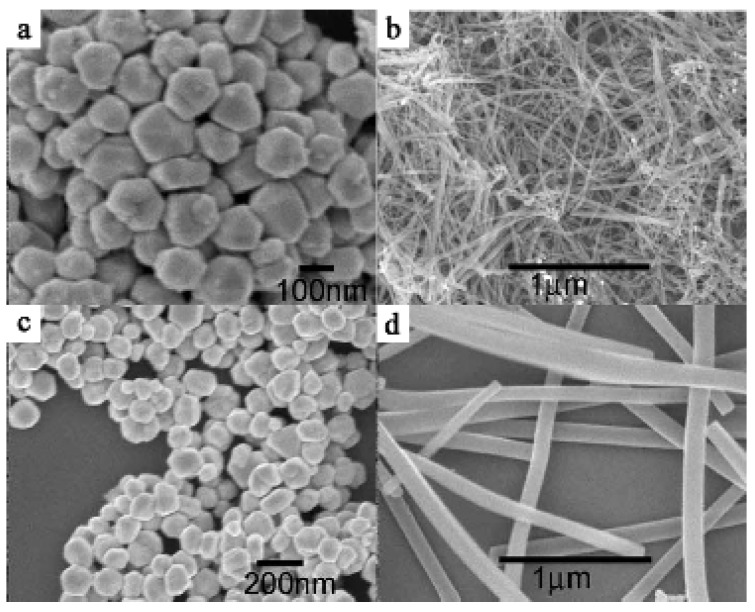
(a) Cu nanoparticles with diameters of about 60−130 nm. (b) Cu•**TCNQ** nanowires prepared by the two-phase method. (c) Ag nanoparticles with diameters of about 80−150 nm. (d) Ag•**TCNQ** nanowires prepared by the two-phase method. [Reproduced with permission from [7] *Nanotechnology* **2007**, *18*, 495704:1–495704:7. ©2007, IOP Publishing Ltd].

Nanowires of Ag•**TCNQ** and Cu•**TCNQ** are also obtained by the mixing technique between a hexane solution of Ag or Cu nanoparticles (diameter in the 60–150 nm range) and an acetonitrile solution of **TCNQ**. The SEM images of Ag•**TCNQ** and Cu•**TCNQ** nanowires and Ag and Cu nanoparticles are shown in Figure 4.

**Figure 5:**
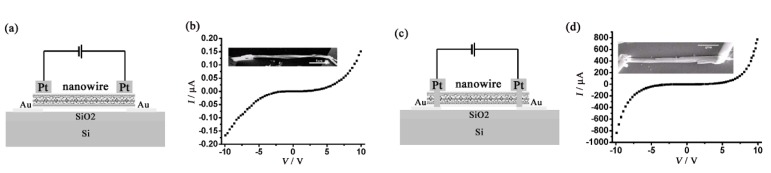
(a) Schematic diagram of the device with contacts made on the side wall of the **TTF**•**TCNQ** helical nanowire and (b) the *I−V* characteristic of the corresponding nanowire in (a). (c) Schematic diagram of the device with contacts made on the ends of the **TTF**•**TCNQ** helical nanowire (open ends were milled with focused ion beam) and (d) the *I−V* characteristic of the corresponding nanowire in (c). [Reproduced with permission from [7] *Nanotechnology* **2007**, *18*, 495704:1–495704:7. ©2007, IOP Publishing Ltd].

The anisotropic electronic transport properties of **TTF**•**TCNQ** helical nanowires are investigated using two different configurations of electrodes as shown in Figure 5(a) and (c). For the device in Figure 5(a), the contacts are made at the side wall of the nanowire with focused ion beam Pt deposition. For this device, conductivities at room temperature are 3.8 × 10^–4^ S cm^–1^ at bias voltages below 1 V, and 1.05 × 10^–2^ S cm^–1^ at bias voltages above 4 V (Figure 5(b)). On the other hand, for the device in Figure 5(c), the Pt electrodes are placed at the open cut at both ends of the nanowire to connect the electrodes and the nanowire together. For this device, the room-temperature conductivity is 0.26 S cm^–1^ at bias voltages below 1 V, and increases to 295 S cm^–1^ at bias voltages above 4 V (Figure 5(d)). These results are well explained by considering that the long axis of the nanowire corresponds to the direction parallel to the **TTF**•**TCNQ** stacking, as also observed in the growth of the single crystal [8], which has large anisotropy in the resistivities (the ratio is 1:160:500 for the TTF•TCNQ intracolumnar, **TTF**•**TTF**/**TCNQ**•**TCNQ** intercolumnar and **TTF**•**TCNQ** stacking directions). The increase in the conductivities at the bias voltages above 4 V is due to the possible electron transport through the energy barrier between the nanowire chains.

#### 2.1.2. Deposition on Stainless Steel Conversion Coating (SSCC) Substrate

**Figure 6:**
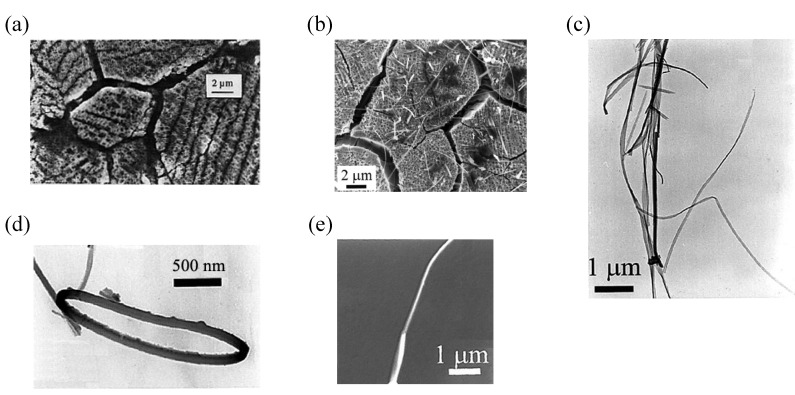
SEM images of (a) native SSCC surface and (b) SSCC surface covered by nanowires, TEM images of (c) a bundle of nanowires and (d) a loop, and (e) 3-D representation of a tapping mode AFM image of a nanowire. [Reproduced with permission from [10] *C. R. Acad. Sci. Paris Ser. IIc* **2000**, *3*, 675–680. ©2000, Académie des sciences / Éditions scientifiques et médicales Elsevier SAS].

**Figure 7:**
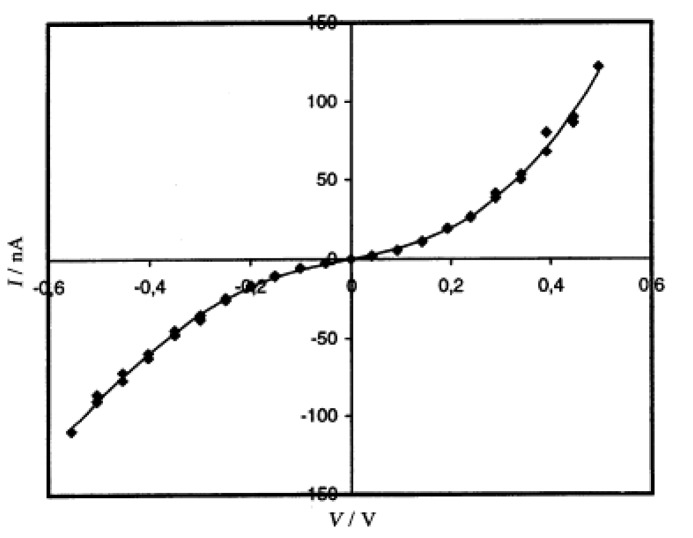
Room temperature current–voltage characteristic of a bundle of nanowires. [Reproduced with permission from [10] *C. R. Acad. Sci. Paris, Ser. IIc* **2000**, *3*, 675–680. ©2000, Académie des sciences / Éditions scientifiques et médicales Elsevier SAS].

SSCCs grow on austenitic stainless steel-sheets through a combined chemical/electrochemical process. The resulting coatings were identified as magnetite and maghemite phases [9]. They have been known to exhibit advanced adsorption properties due to their fractal-like nano-structured surface (Figure 6(a)) and applied to fix dyes or to improve the adherence of further coatings. The **TTF**•**TCNQ** nanowires are prepared by the successive immersion of this SSCC in CH_3_CN solutions (10^–2^ M) of **TTF** and **TCNQ** at room temperature [10]. Adsorption of **TTF** was realized at first. Then, immersion of the **TTF**-coating surface in the **TCNQ** solution resulted in the formation of nanowires and few platelets. As shown by the SEM image in Figure 6(b), the nanowires are anchored on the SSCC surface, and some of them bridge the grain boundaries of the conversion coating (boundary separation = 1–2 μm). The nanowires were easily separated from the substrate and used for TEM, AFM and *I*–*V* measurements. From the TEM observation in Figure 6(c), the nanowires are actually ribbons having an average thickness of 20 nm and a width between 20 and 200 nm. These ribbons are 20 μm long, and occasionally produce small loops (Figure 6(d) and Figure 6(e)). The *I*–*V* characteristics are measured for a bundle of fibers deposited with a micropipette on a metal–insulator–metal nano-junction (Figure 7). As the fibers are not parallel to the electrodes, the conductivity deduced from the curve is dominated by the perpendicular conductivity. The room-temperature conductivity is about 1 S cm^–1^, and the shape of the *I*–*V* curve is similar to that observed for single crystals of **TTF**•**TCNQ** deposited on an alkali halide surface and studied by use of scanning tunneling microscopy [11], a method that also gives a characterization perpendicular to the wires.

**Figure 8:**
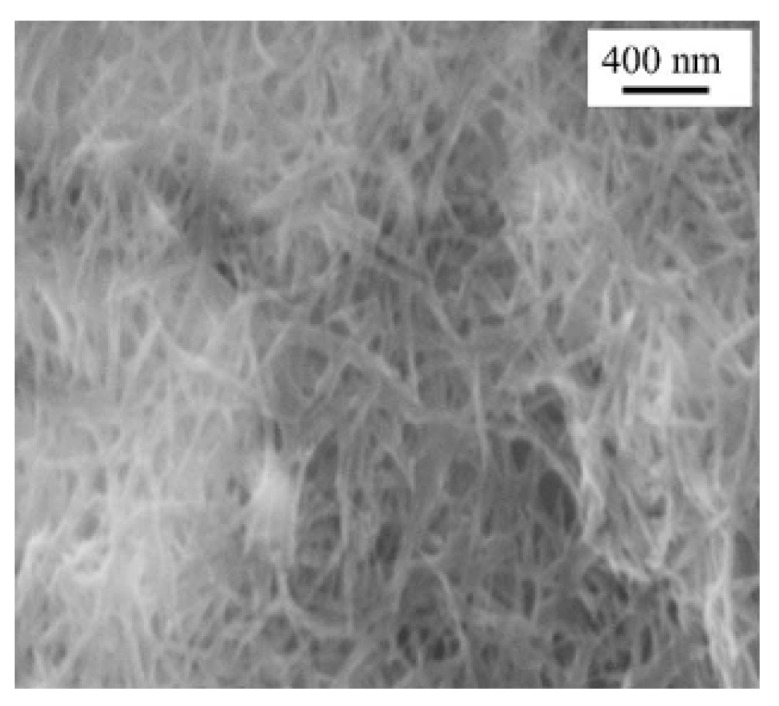
SEM image of **TTF**•[Ni(dmit)_2_]_2_ nanowires on SSCC. [Reproduced with permission from [12] *J. Solid State Chem.* **2002**, *168*, 438–443. ©2002, Elsevier Science (USA)].

**Figure 9:**
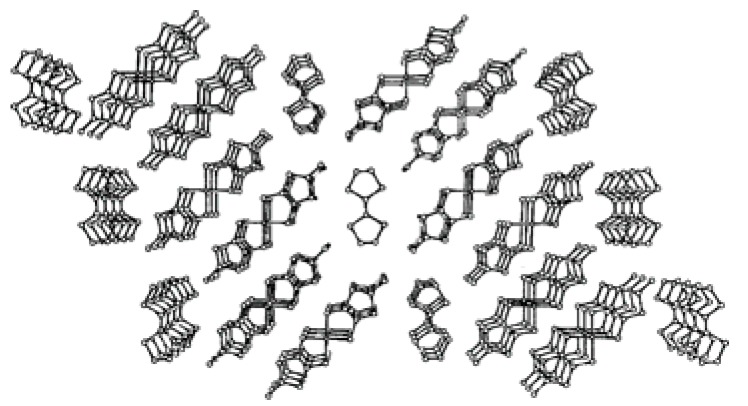
View of the **TTF**•[Ni(dmit)_2_]_2_ structure along the [010]–stacking axis. [Reproduced with permission from [12] *J. Solid State Chem.* **2002**, *168*, 438–443. ©2002, Elsevier Science (USA)].

The above dipping process on the SSCC substrate with nanopores also gave nanowires of **TTF**•[Ni(dmit)_2_]_2_ [12]. When the substrate is first dipped in a CH_3_CN solution of NBu_4_•[Ni(dmit)_2_], the nanopores of the surface uniformly adsorb the solution. The adsorbed amount of solution can be increased if the substrate is place under vacuum before dipping. The NBu_4_•[Ni(dmit)_2_]-filled surface is again dipped in a CH_3_CN solution of (**TTF**)_3_•(BF_4_)_2_. At this time, the reaction occurs between the two components and the nanowires of **TTF**•[Ni(dmit)_2_]_2_ grow up on the surface. The SEM image of the nanowires on the SSCC substrate is shown in Figure 8. The diameter of these nanowires is 50 to 150 nm. Some areas are much more covered by the nanowires than others. Comparison of Raman spectra of the nanowires and single crystals shows that the nanowires have the same molecular composition as the single crystals, whose structure consists of segregated stacks of TTF and [Ni(dmit)_2_] units (Figure 9) from comparison of Raman spectra between the nanowires and single crystals. The nanowires were only located on such a small part of the substrate surface that they could not be removed from the substrate even by use of a micropipette. Moreover, the conductivity of the nanowires located on the substrate could not be measured due to the conducting character of substrate.

#### 2.1.3. Electrochemical Deposition on Native Silicon Wafer Electrode

**Figure 10:**
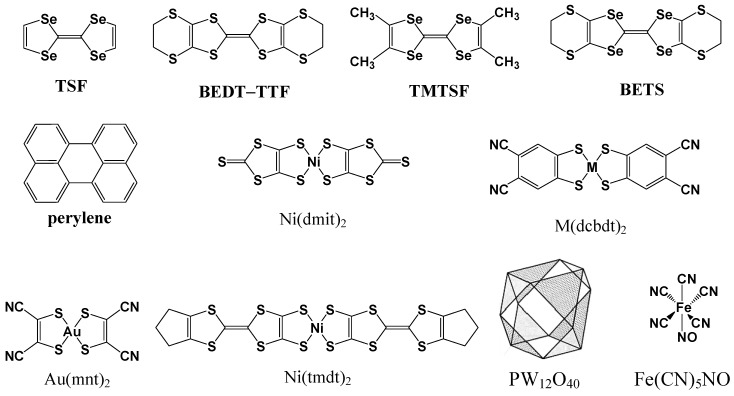
The chemical structures of donor molecules (**TSF**, **BEDT–TTF**, **TMTSF**, **BETS**, and **perylene**) and anions (Ni(dmit)_2_^–^, M(dcbdt)_2_^–^, Au(mnt)_2_^–^, Ni(tmdt)_2_^–^, PW_12_O_40_^3–^, and Fe(CN)_5_NO^–^) used in electrochemical oxidation.

Electrochemical oxidation of **TTF**, bis(ethylenedithio)–TTF (**BEDT–TTF**), and tetramethyl- and bis(ethylenedithio)-tetraselenafulvalenes (**TSF**) (**TMTSF** and **BETS**), **perylene**, NMe_4_•[Ni(tmdt)_2_], NBu_4_•[M(dcbdt)_2_] (M = Co, Ni, Au), *etc.*, in the presence of several supporting electrolytes, NBu_4_•[Ni(dmit)_2_], NBu_4_•ClO_4_, NBu_4_•[M(dcbdt)_2_] (M = Co, Ni, Au), (NEt_4_)_3_•[PW_12_O_40_], NBu_4_•[Fe(CN)_5_NO], NBu_4_•[Au(mnt)_2_] *etc.* (Figure 10) was carried out using a native silicon wafer as an electrode. Conductors obtained by the above reactions were typically deposited on the surface of silicon wafer with morphologies of grains, flakes, blocks, needles, plates, sheets and films. Very occasionally, micro/nano-wires were formed.

**Figure 11:**
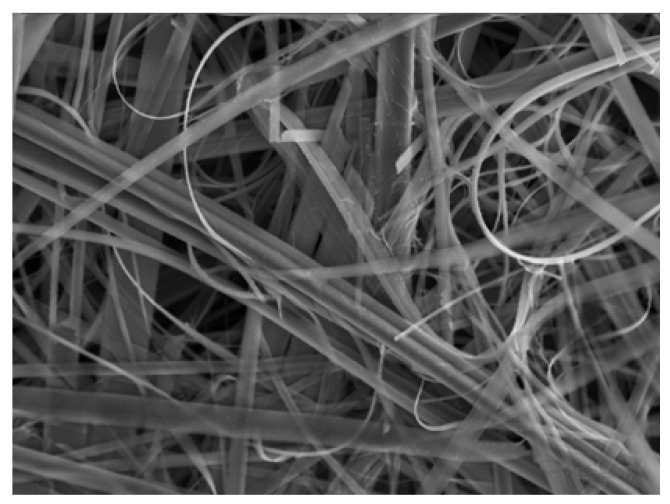
SEM image of **TTF**•[Ni(dmit)_2_]_2_. [Reproduced with permission from [6] *J. Phys.: Condens. Matter.* **2008**, *20*, 184012:1–184012:10. ©2008, IOP Publishing Ltd].

**Figure 12:**
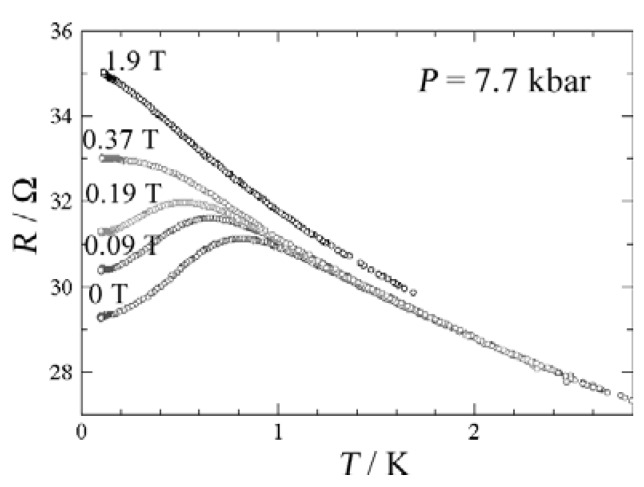
Temperature dependence of the resistance of **TTF**•[Ni(dmit)_2_]_2_ at *P* = 7*.*7 kbar, in the superconducting domain, for different applied magnetic fields ranging from 0 to 1.9 T. [Reproduced with permission from [6] *J. Phys.: Condens. Matter.* **2008**, *20*, 184012:1–184012:10. ©2008, IOP Publishing Ltd].

When a CH_3_CN solution of **TTF** and NBu_4_•[Ni(dmit)_2_] was subjected to electrolysis by applying a high current density of about 6.2 μA cm^–2^, bundles made of microwires with width of 1–2 μm and length of 20–60 μm were deposited on the silicon wafer [13]. The SEM image is shown in Figure 11. This deposit showed the same X-ray diffraction pattern, X-ray photoelectron spectrum and Raman spectrum as those of the single crystals of **TTF**•[Ni(dmit)_2_]_2_, which exhibits a metallic behavior down to 4 K, and a transition to a superconducting state at 1.6 K under a pressure of 7 kbar. Resistivity measurement of the bundles was performed between ambient pressure and 7.7 kbar. Whatever the pressure applied, the resistivity is nearly constant in the temperature range of 100 to 300 K, and significantly increases below 100 K, being characteristic of a semiconducting behavior. Between 100 and 300 K, inter-bundle contacts do not affect too much the overall conducting behavior. The room-temperature conductivities are 9 S cm^–1^ at ambient pressure and 24 S cm^–1^ under 7.7 kbar. A broad drop of the resistivity is observed below 0.8 K under 7.7 kbar (Figure 12), but the transition observed is incomplete, presumably due to the contribution of grain boundaries. The occurrence of superconductivity is confirmed by applying magnetic field perpendicular to the silicon substrate plane. The drop of the resistivity gradually becomes smaller with increasing magnetic field, and at 1.9 T the transition completely disappears. The critical magnetic field is estimated to be 0.45 T, a lower value compared with that (2.5 T) of the single crystals due to the inter-bundle resistivity contribution [14,15].

**Figure 13:**
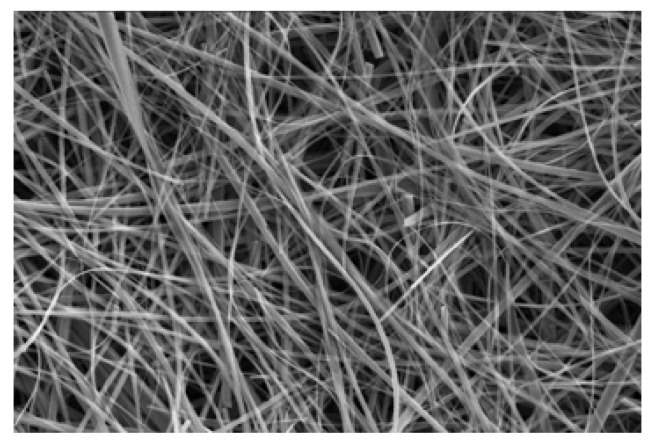
SEM image of (**TMTSF**)_5_•[Co(dcbdt)_2_]_4_. [Reproduced with permission from [6] *J. Phys.: Condens. Matter* **2008**, *20*, 184012:1–184012:10. ©2008, IOP Publishing Ltd].

The electrolysis of a CH_2_Cl_2_ solution of **TMTSF** and NBu_4_•[Co(dcbdt)_2_] gave a deposition of microwires on silicon wafer [3]. The SEM image is shown in Figure 13. The microwires are <3 μm wide and >100 μm long. The molecular formula was determined as (**TMTSF**)_5_•[Co(dcbdt)_2_]_4_ by X-ray photoelectron spectroscopy (XPS). The Raman spectral measurement indicates that the charge residing on the **TMTSF** molecules is +0.8 based on a linear relationship between the C=C stretching frequency due to the **TMTSF** molecule and the charge and this result is in good agreement with the stoichiometry determined by XPS. The room-temperature conductivity of the microwires is comparatively high (2 S cm^–1^), but the conducting behavior is semiconducting and the activation energy is 56 meV.

**Figure 14:**
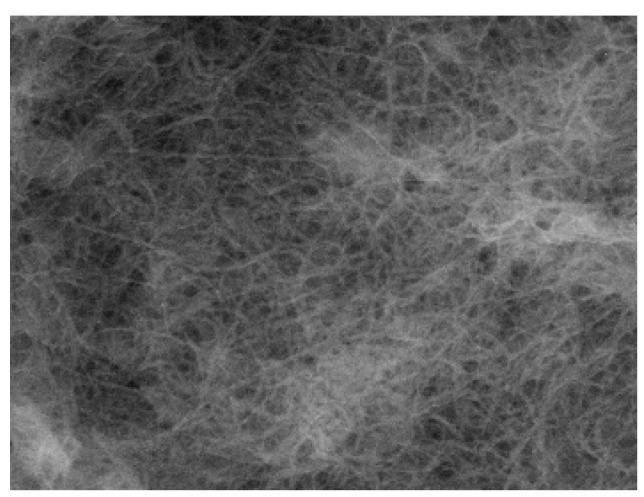
SEM image of (**perylene**)_2_•[Au(mnt)_2_]. [Reproduced with permission from [6] *J. Phys.: Condens. Matter* **2008**, *20*, 184012:1–184012:10. ©2008, IOP Publishing Ltd].

The above two molecular conductor wires were of micrometer size. The actual nano-size molecular conductor wires were obtained with (**perylene**)_2_•[Au(mnt)_2_] [16]. A CH_2_Cl_2_ solution of **perylene** and NBu_4_•[Au(mnt)_2_] was electrochemically oxidized at constant current density of 0.30 μA cm^–2^ using a silicon wafer electrode. A black-colored deposit made of nanowires was obtained on the silicon anode, as shown by the SEM image in Figure 14. The diameter of the nanowires is 35–55 nm, and the length is about 10 μm. The 2:1 stoichiometry of **perylene** and [Au(mnt)_2_] units was confirmed by the elemental analysis of a sample scratched from the silicon substrate. The conductivity of the nanowires deposited on the silicon wafer is about 0.02 S cm^–1^ at room temperature, and follows a thermally-activated semiconducting behavior with activation energy of 88 meV. The room-temperature conductivity of the nanowires is lower by about 10^4^-times than that of the single crystals. The main causes are considered to be the random orientation of the nanowires on the substrate and the resistive inter-nanowire contacts.

#### 2.1.4. Electrochemical Deposition on Platinum Electrode

**Figure 15:**
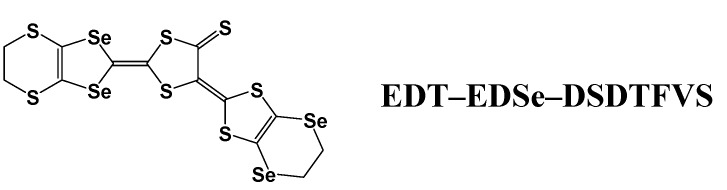
The chemical structure of ethylenedithio-ethylenediseleno-diselenadithiafulvalenothioquinone-1,3-dithiolemethide (**EDT–EDSe–DSDTFVS**).

An example of the formation of molecular conductor nanowires by use of a conventional platinum rod electrode was found by our group [17]. A bent donor molecule with a relatively large arc, ethylenedithiodiselenadithiafulvalenothioquinone-1,3-ethylenediselenadithiole (**EDT–EDSe– DSDTFVS**) (Figure 15) and its related derivatives, tend to form highly one-dimensional stacks in the radical cation salts [18]. When **EDT–EDSe–DSDTFVS** was electrochemically oxidized in a PhCl/EtOH containing a supporting electrolyte, NBu_4_•FeCl_4_ with a fairly high current of 1.0 μA at 30 °C, very thin and long micro/nano-wires were formed on the surface of a platinum rod electrode. The SEM images of the micro/nano-wires are shown in Figur 16(a)−(c). The maximum length is >3 cm with good flexibility that can be wrapped to a circle without breaking the micro/nano-wires. The width and thickness are in the micrometer and nanometer scales, respectively. At a temperature lower than 20 °C, a larger amount of micro/nano-wires covered the electrode surface. The length of the micro/nano-wires was reduced, but the width and thickness remained almost the same. Based on the SEM–EDX measurement (Figure 17(a)), the micro/nano-wires are proved to be the FeCl_4_^–^ salt of **EDT–EDSe–DSDTFVS**, which has a molecular composition of (**EDT–EDSe–DSDTFVS**)_2_•FeCl_4_ from the weight ratio of S, Cl, Fe and Se atoms (Figure 17(b)) (S:Cl:Fe:Se = 14.37:5.03:1:8.30, which is well consistent to the calculated one of 14:4:1:8).

**Figure 16:**
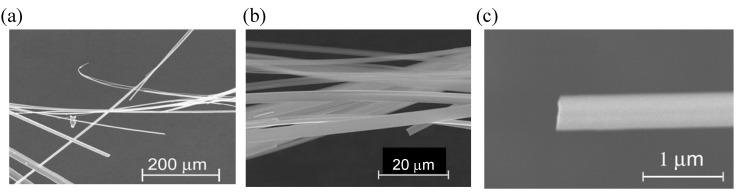
SEM images of (a and b) bundle and (c) single micro/nano-wires of (**EDT−EDSe−DSDTFVS**)_2_•FeCl_4_. [Reproduced with permission from [17] *Chem. Mater.* **2009**, *21*, 5569–5571. ©2009, American Chemical Society].

Under electrochemical conditions applying a smaller current of 0.2 μA and a higher temperature of 45 °C, very thin and rectangular platelet crystals were instead obtained. The unit cell parameters are determined to be *a* = 7.187(7) Å, *b* = 17.17(1) Å and *c* = 43.91(4) Å, which correspond to the width, length and thickness of the single crystals. The crystal structure is not solved because of the too small thickness. The powder X-ray diffraction patterns for the micro/nano-wires and the single crystals are almost the same, suggesting that the micro/nano-wires have the same stacking structure to that of the single crystals.

The AFM measurement of a single micro/nano-wire was performed. From the AFM image in Figure 18(a) the surface of the wire shows stepwise morphology. The height of each step is around 4–6 nm, suggesting that the micro/nano-wire is intrinsically composed of nanowires with the thickness of *ca.* 5 nm. The nanowires adhered to each other along the crystal *c*–axis, and cause the disorder along this direction. The thickness of the nanowire is very close to the length (4.4 nm) of the crystal *c*–axis. By careful comparison of the unit cell parameters of (**EDT–EDSe–DSDTFVS**)_2_•FeCl_4_ with those of the radical cation salts of the bent donor molecules related to **EDT–EDSe–DSDTFVS**, the *c*–axis of (**EDT–EDSe–DSDTFVS**)_2_•FeCl_4_ should correspond to the donor-anion direction. The *a*– and *b*–axes are along the side-by-side and face-to-face arrays of donor molecules, respectively. The schematic depiction of the arrays of donor molecules and FeCl_4_^–^ ions in micro/nano-wires is shown in Figure 18(b).

**Figure 17:**
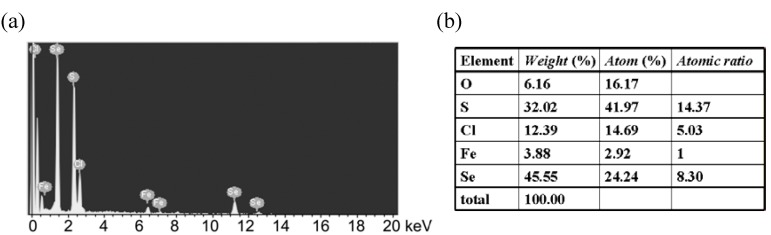
(a) SEM−EDX spectrum of (**EDT−EDSe−DSDTFVS**)_2_•FeCl_4_ and (b) analysis results.

The crystal growth is a competitive process between the thermodynamic and kinetic factors. In the early stage a nucleation occurs and forms a unit cell of (**EDT–EDSe–DSDTFV**)_2_•FeCl_4_. Under the relatively high temperature (45 °C) and low current (0.2 μA), the thermodynamic factors play the dominant role. The donor molecules and FeCl_4_^–^ ions diffuse from the bulk phase and adsorb onto each direction of the unit cell. Accordingly, the bulk crystals are formed. On the other hand, when a lower temperature (30 °C) and a higher current (1.0 μA) are applied to the electrochemical oxidation, the diffusion rates of donor molecules and FeCl_4_^–^ ions are suppressed. In this case, the kinetic factors control the crystal growth. Driven by the large current density along the face-to-face array, the donor molecules are quickly electrochemically oxidized and adsorb to this prior direction. The FeCl_4_^–^ ions migrate to combine with (**EDT–EDSe–DSDTFVS**)^+•^ species, giving rise to the formation of ultralong micro/nano-wires of (**EDT–EDSe–DSDTFVS**)_2_•FeCl_4_. Because the donor–anion interactions are relatively weak in the alternately-layered structures of radical cation salts, the rate of crystal growth along this direction is the lowest among the three directions of the crystal. Consequently, only one unit cell was generated along this direction during the crystal growth. At temperatures below 20 °C, the solubility of (**EDT–EDSe–DSDTFVS**)_2_•FeCl_4_ was further decreased. The nucleation became more rapid to cover the surface of the platinum rod electrode by the suppression of the further elongation of the micro/nano-wires. The size of the micro/nano-wires can be tuned by controlling the temperature and/or time for electrocrystallization.

The temperature-dependent resistivity of the several pieces of microwires was measured along the long axis. A semiconducting behavior was observed. However, the room-temperature conductivity was considerably high, around 40–60 S cm^–1^. The activation energy for electrical conduction was about 10 meV. Along with their good flexibility, the highly conducting property implies that the present micro/nano-wires are expected as molecular wires bridging between two nano-size electrodes in a nano-size molecular transistor. These conducting properties are comparable to those of the corresponding single crystals exhibiting a semiconducting behavior with the room-temperature conductivity of 30 S cm^–1^ and activation energy of about 20 meV. The slight difference of the conducting properties between the microwires and the single crystals may be due to the different degree of crystallinity, where the former should have fewer defects than the latter.

**Figure 18:**
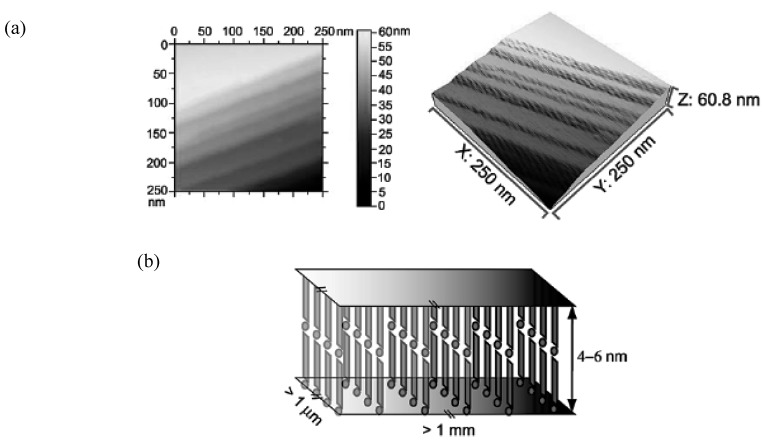
(a) AFM images of a single nanowire of (**EDT–EDSe–DSDTFVS**)_2_•FeCl_4_, where X, Y and Z correspond to the length, width and thickness, and (b) schematic depiction of the molecular arrays in the nanowire. [Reproduced with permission from [17] *Chem. Mater*. **2009**, *21*, 5569–5571. ©2009, American Chemical Society].

**Figure 19:**
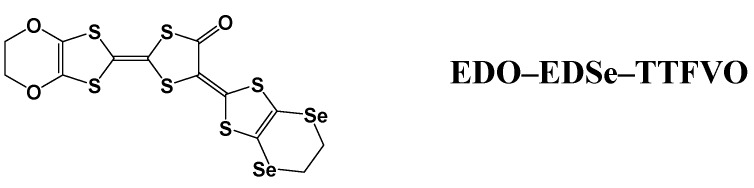
The chemical structure of ethylenedioxy-ethylenediseleno-tetrathiafulvaleno-quinone-1,3-dithiolemethide (**EDO–EDSe–TTFVO**).

Based on the similar concept, the electrochemical oxidation was performed in an 1,2-dichloroethane (DCE) solution containing NBu_4_FeCl_4_ by applying the constant current of 1.0 μA at –5 °C for another bent donor molecule with a large arc, ethylenedioxy- tetrathiafulvalenoquinone-1,3-ethylenediselenadithiolemethide (**EDO–EDSe–TTFVO**) (Figure 19). Instead of ultralong micro/nano-wires as expected, microwires of (**EDO–EDSe–TTFVO**)_2_•FeCl_4_•(DCE)_0.5_ were obtained. Of course, this method can be applied to the other donor molecules which show the strong tendency to stack along a specific direction.

#### 2.1.5. Electrochemical Deposition on Nano-Size Electrode in Solution

**Figure 20:**
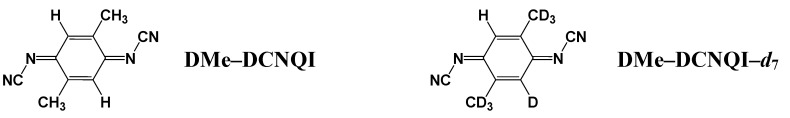
The chemical structures of 2,5-dimethyl-*N*,*N’*-dicyanoquinodiimine (**DMe–DCNQI**) and its *d*_7_ derivative (**DMe–DCNQI–*d*_7_**).

**Figure 21:**
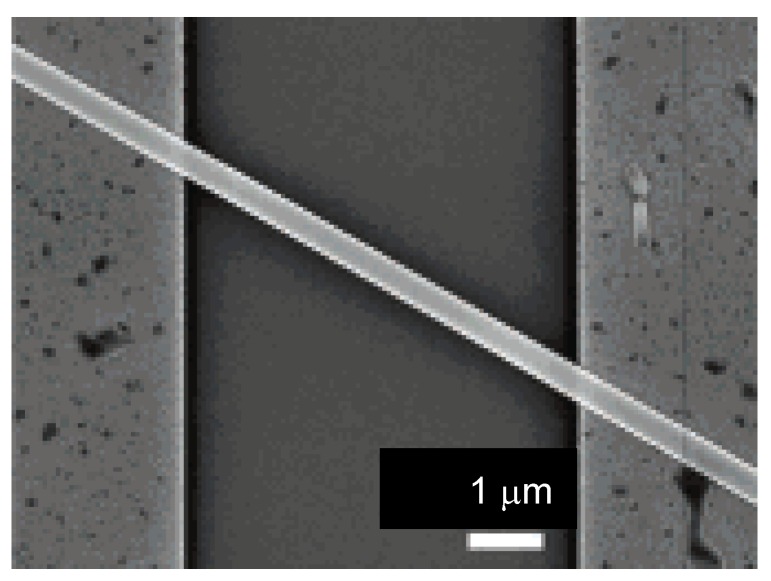
Micro/nano-wires of (**DMe–DCNQI**)_2_•Ag bridging two Au electrodes. [Reproduced with permission from [19] *J. Am. Chem. Soc*. **2006**, *128*, 700–701. ©2006, American Chemical Society].

Chemical and electrochemical methods to get molecular conductor micro/nano-wires directly on silicon substrate were developed [19]. In the chemical method Ti (2 nm), Au (15 nm), and Ag (30 nm) are deposited in this order onto SiO_2_ (200 nm)/doped–Si substrate to form a nano-size electrode. The substrate is immersed in a CH_3_CN solution of an acceptor molecule, 2,5-dimethyl-*N*,*N*’-dicyanoquinodiimine (**DMe–DCNQI**) (Figure 20) in a glass cell, and the subsequent redox reaction between Ag and **DMe–DCNQI** results in the micro/nano-wire of (**DMe–DCNQI**)_2_•Ag large enough to bridge the gap between the nano-size electrodes (Figure 21). The size of the micro/nano-wires is 1–100 μm in length and 100 nm–10 μm in width and thickness. Although the direction of the crystal growth is initially random, growth becomes preferentially parallel to the substrate surface due to the mechanical instability of standing crystals under solvent flow across the substrate and by the rapid drop in concentrations of Ag^+^ and [**DMe–DCNQI**]^–•^ ions away from the electrode surface. After removal of the substrate from the solution and drying, the electrodes are cut by laser ablation to form a circuit including the micro/nano-wire.

**Figure 22:**
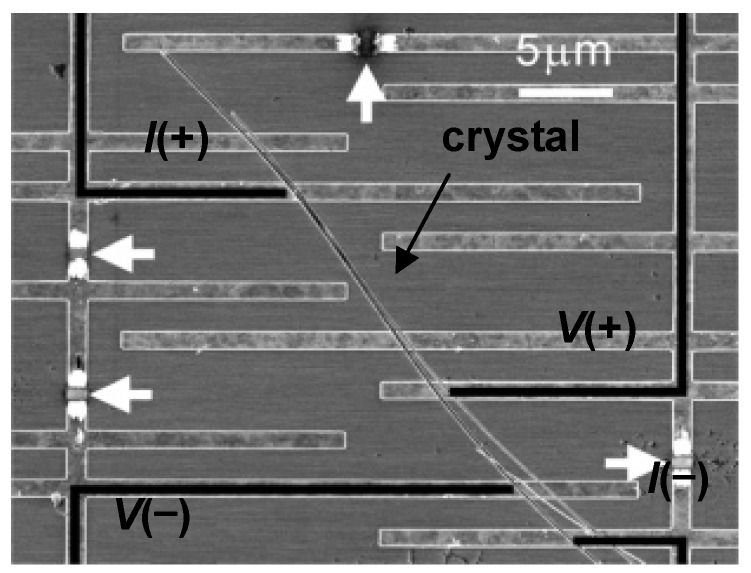
SEM image of four-probe circuit on a (**DMe–DCNQI**)_2_•Cu micro/nano-wire of 100 nm in width and thickness and 60 *μ*m in length. Electrodes were cut at the points indicated by white arrows. [Reproduced with permission from [19] *J. Am. Chem. Soc*. **2006**, *128*, 700–701. ©2006, American Chemical Society].

On the other hand, in the electrochemical method the electrode is prepared by deposition of Ti (2 nm) and Au (10 nm) onto the SiO_2_/doped-Si substrate, and the substrate is also immersed in a CH_3_CN solution of **DMe–DCNQI** and a supporting electrolyte, AgClO_4_. The cathodic probe is attached to the Au electrode on the substrate, while the anodic probe is attached into contact with the doped Si gate electrode. The micro/nano-wire of the corresponding Cu salt, (**DMe–DCNQI**)_2_•Cu is also obtained by the same procedure using Cu instead of Ag (chemical method) or using (NEt_4_)_2_•CuBr_4_ instead of AgClO_4_ (electrochemical method). The SEM of four-probe circuit on a (**DMe–DCNQI**)_2_•Cu nanowire with 100 nm in width and thickness, and 60 μm in length is shown in Figure 22.

**Figure 23:**
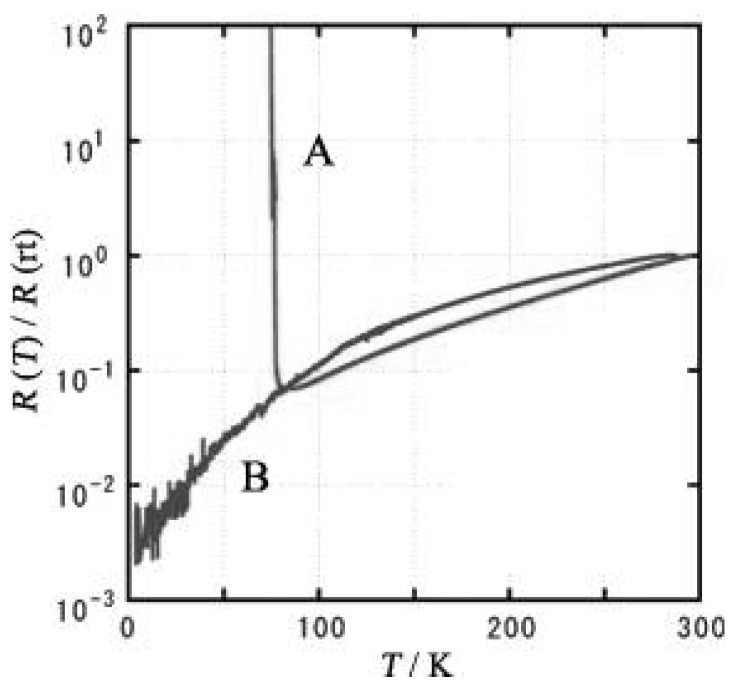
Temperature dependence of normalized resistivity for single crystal (A) and micro/nano-wire (B) of (**DMe–DCNQI–*d*_7_**)_2_•Cu. [Reproduced with permission from [19] *J. Am. Chem. Soc*. **2006**, *128*, 700–701. ©2006, American Chemical Society].

The bulk crystal of (**DMe–DCNQI**)_2_•Ag is known to exhibit a rapid increase in resistivity below 100 K. A similar behavior is obtained also for the single micro/nano-wire, which however exhibits no weakly metallic behavior near room temperature as observed in the bulk crystal, probably due to the influence of contact resistance. On the other hand, the conducting behavior of the single nanowire of (**DMe–DCNQI–*d*_7_**)•Cu (**DMe–DCNQI–*d*_7_** is a derivative of **DMe–DCNQI** (Figure 20), whose seven hydrogen atoms are replaced by deuterium atoms) presents a striking contrast to that of the bulk crystal, as shown in Figure 23. The nanowire exhibits a continuous decrease in resistivity with decreasing temperature without metal–insulator (M–I) transition at 80 K, which occurs in the bulk crystal. The metallic character is kept down to low temperature for the nanowire. In this measurement the contact resistance is very low (*ca.* 1 kΩ) in spite of the small contact area (*ca.* 0.1 μm^2^).

**Figure 24:**
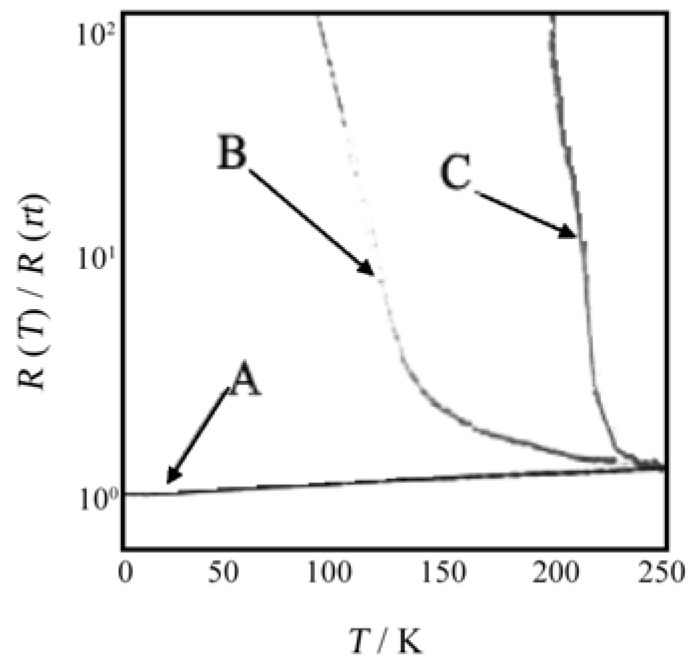
Temperature dependence of the normalized resistivities for (A) (**TMTSF**)_2_•PF_6_, (B) *α*–(**BEDT–TTF**)_2_•I_3_, and (C) (**EDO–TTF**)_2_•PF_6_.

**Figure 25:**
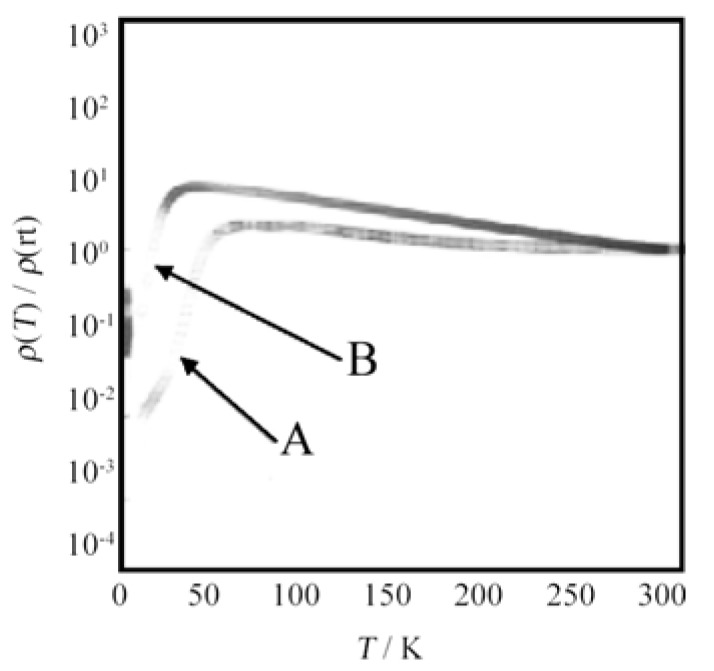
Temperature dependence of the normalized resistivities for the (A) bulk and (B) nano (thickness of about 100 nm, and width of about 50 μm) crystals of *κ*−(**BEDT–TTF**)_2_•Cu[N(CN)_2_]Br.

The electrochemical method using the above nano-size electrode was also applied to the preparation of micro/nano-crystals of radical cation salts of **TTF** derivatives, **BEDT–TTF**, **TMTSF** and ethylenedioxytetrathiafulvalene (**EDO–TTF**), *α*–(**BEDT–TTF**)_2_•I_3_, (**TMTSF**)_2_•PF_6_, (**EDO–TTF**)_2_•PF_6_ and *κ*–(**BEDT–TTF**)_2_•Cu[N(CN)_2_]Br [20]. The micro/nano-crystals were placed on the nano-size electrodes for the four-probe electrical conductivity measurement. As can be seen from the temperature dependence of the normalized resistivities in Figure 24, (**TMTSF**)_2_•PF_6_ exhibits a decrease of the resistivity down to 4 K without any M–I transition. This result is in sharp contrast to that of the bulk crystal, in which M–I transition occurs at 12 K. A monotonical increase of resistivity from room temperature is observed for the nano-crystal of *α*–(**BEDT–TTF**)_2_•I_3_, while the bulk crystal exhibits M–T transition at 135 K. For both the nano- and bulk-crystals of (**EDO–TTF**)_2_•PF_6_ M–T transition is observed, but the transition becomes lower by 20 K for the nano-crystal than for the bulk crystal. The temperature dependence of the normalized resistivities for the (A) bulk and (B) nano crystals of *κ*–(**BEDT–TTF**)_2_•Cu[N(CN)_2_]Br is shown in Figure 25. The bulk crystal becomes a superconductor at 11 K via metal-to- semiconductor (M–S) transition near 60 K. On the other hand, for the nano-crystal the similar M–S transition occurs near 30 K, but there is no drop in the resistivity in the lower temperature range.

Several causes are conceivable for the different conducting behavior between the single nano- and bulk-crystals of (**DMe–DCNQI–d_7_**)•Cu and radical cation salts of **TTF** derivatives. The difference of thermal contraction rate and work function between the electrode and the molecular conductors, which results in partial doping of the crystal, may be important factors. More details of the size effect, *i.e.*, the surface/volume ratio of the crystal are required to clarify the origin.

#### 2.1.6. Electrochemical Deposition on Nano-size Electrode in Vacuum Evaporation

**Figure 26:**
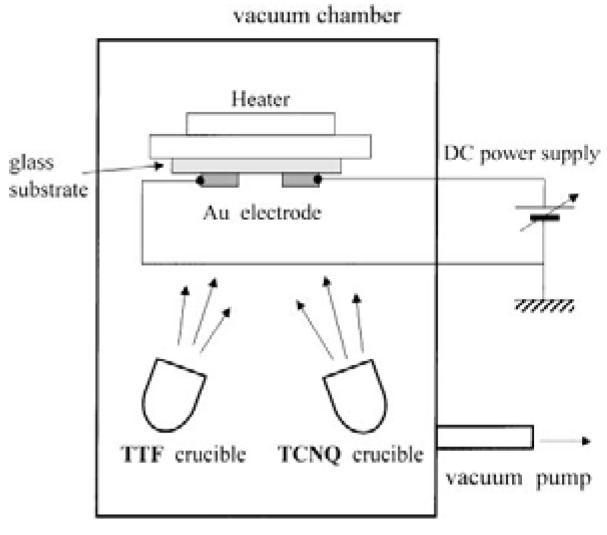
Schematic diagram of the applied electric field deposition method. [Reproduced with permission from [21] *Jpn. J. Appl. Phys.* **2003**, *42*, 2488–2491. ©2003, Japanese Society of Applied Physics].

**Figure 27:**
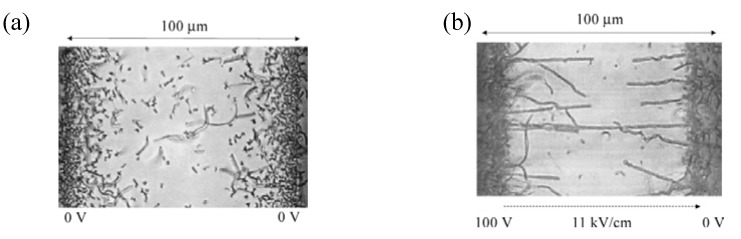
Optical microscopy images of (a) **TTF**•**TCNQ** grains formed by the co-evaporation technique with zero electric field at 42 °C and of (b) **TTF**•**TCN**Q wires grown at 42 °C under high electric field of 11 kV/cm (110 V). The electrode gap is 100 μm. [Reproduced with permission from [21] *Jpn. J. Appl. Phys.* **2003**, *42*, 2488–2491. ©2003, Japanese Society of Applied Physics].

In the foregoing section a useful preparation method of molecular conductor nanowires was described, which uses electrochemical reaction of donor or acceptor molecules with nano-size electrodes in solution. The corresponding *dry* method, the co-evaporation of **TTF** and **TCNQ** with electric field was developed [21]. In this method, two Au electrodes on the glass substrate are settled with a separation distance of 20 or 100 μm, and the substrate is kept at 40–45 °C (Figure 26) [22,23]. A DC electric field of 6–35 kV cm^–1^ was applied between the two electrodes during the co-evaporation of **TTF** and **TCNQ**. The surface morphology of **TTF**•**TCNQ** was observed using an optical microscope. Figure 27(a) shows the optical microscopy image of **TTF**•**TCNQ** obtained with zero electric field (the electrode gap is 100 μm) at 42 °C. Randomly-oriented microcrystals of **TTF**•**TCNQ** are formed on the glass substrate, particularly around the electrodes. When the average electric field of 11 or 35 kV cm^–1^ is applied between the electrodes under the same conditions as above, highly-oriented wire-like **TTF**•**TCNQ** crystals are grown from the electrodes and aligned along the electric field. Some of a pair of the wires make connection at their tops (Figure 27(b)). In general, **TTF**•**TCNQ** wires grown from the high-voltage electrode tend to be longer than those from the zero-voltage electrode. Increased amount of the wires bridged between the electrodes can be achieved by optimizing the growth conditions such as electrode separation distance, electric field, and growth temperature.

**Figure 28:**
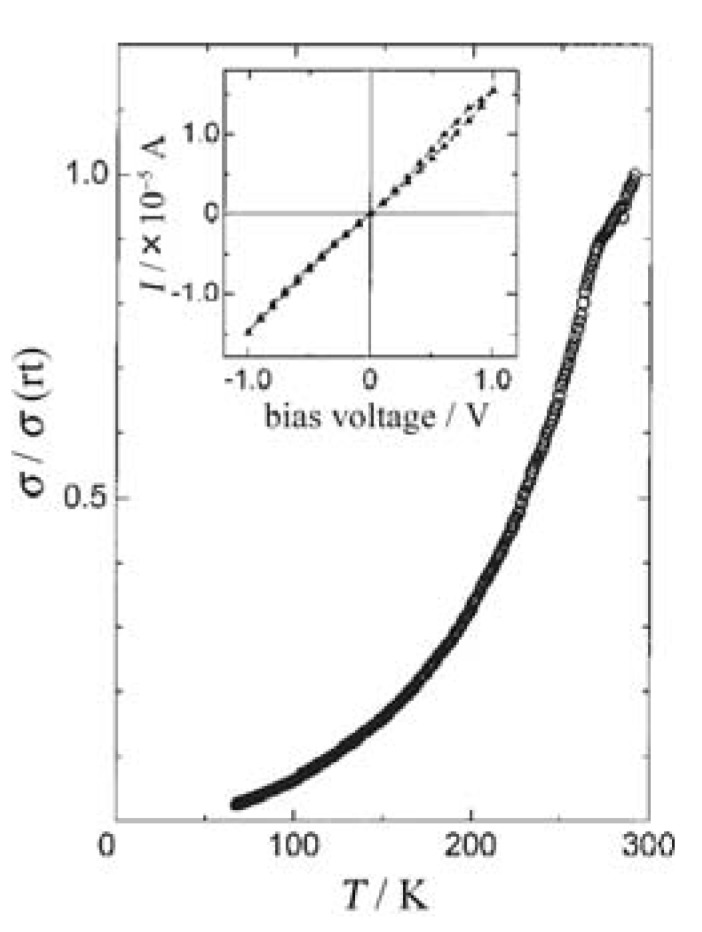
Temperature dependence of normalized conductivity in **TTF**•**TCNQ** wires. Inset: the *I–V* curve of connected **TTF**•**TCNQ** wires. [Reproduced with permission from [21] *Jpn. J. Appl. Phys.* **2003**, *42*, 2488–2491. ©2003, Japanese Society of Applied Physics].

Temperature dependence of normalized conductivities (*σ*/*σ*_RT_: *σ*_RT_ is room-temperature conductivity) and current (*I*)–bias voltage (*V*) curve for the connected **TTF**•**TCNQ** wire are shown in Figure 28 and the inset, respectively. The almost linear *I*–*V* curve indicates the Ohmic junction between the Au electrode and **TTF**•**TCNQ** wire. The *σ*/*σ*_RT_ decreases with decreasing temperature, and this behavior is like a semiconductor with an activation energy of 0.02–0.1 eV. This result is in sharp contrast to that for the bulk **TTF**•**TCNQ** single crystal exhibiting metallic behavior above 53 K. One conceivable cause of this behavior is that the wire is not pure **TTF**•**TCNQ**, but partly contains a nonstoichiometric component of (**TTF**)_1–δ_•**TCNQ** with a high-doped semiconducting behavior.

Interestingly, different growth of **TTF**•**TCNQ** wires from the cathode and anode was confirmed by AFM potentiometry. The wires grown from the cathode are good conductors with *σ*_RT_ > 100 S cm^–1^, while those from the anode are semiconductors with *σ*_RT_ ~ 0.3 S cm^–1^. Moreover, the connection point of the two wires from the cathode and anode shows extremely high resistivity (~2.8 × 10^6^ Ω). Obviously, metallic and semiconducting areas co-exist in the **TTF**•**TCNQ** wires obtained by this method. Accordingly, the tips of the two wires independently grown from the cathode and anode are hard to connect with keeping uniform molecular stacking at the contact point, where resistivity becomes too large. As evidenced from the high conductivity of the wires grown from the cathode, the long axis of the wire corresponds to the stacking direction of **TTF** and **TCNQ** molecules. However, there remains as a serious problem whether or not wires with completely metallic **TTF**•**TCNQ** composition can be prepared by this method.

### 2.2. Template-Assisted Method

#### 2.2.1. Electrochemical Reaction in the Presence of Template Molecule Coordinated with Counteranion

**Figure 29:**
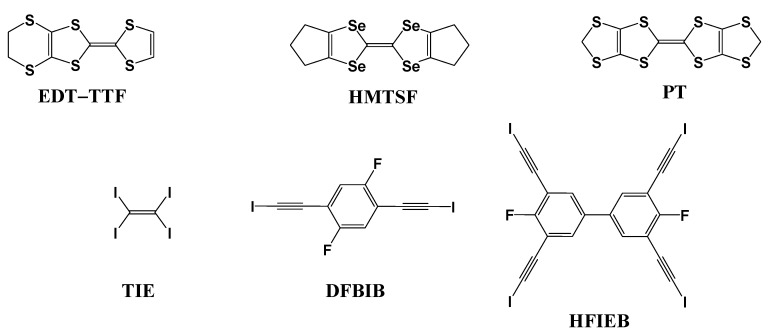
The chemical structures of donor molecules (**EDT–TTF**, **HMTSF**, and **PT**) and iodine-containing neutral molecules (**TIE**, **DFBIB**, and **HFIEB**).

**Figure 30:**
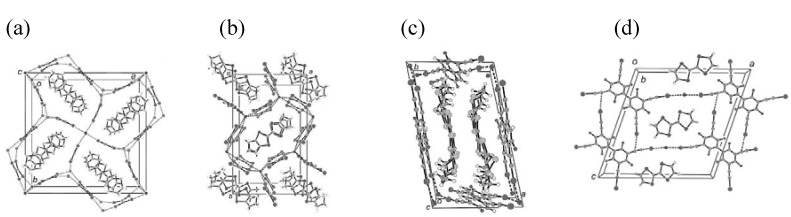
Crystal structures for (a) **1**, (b) **2**, (c) **3**, and (d) **4**. The dotted lines denote the “halogen bond” based on Lewis acidity of the neutral iodine atoms. [Reproduced with permission from [27] *ACSNano* **2008**, *2*, 143–155. ©2008, American Chemical Society].

**Figure 31:**
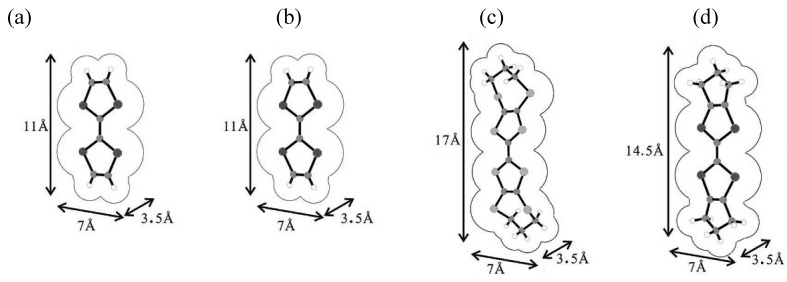
The van der Waals outlines are drawn for (a) **EDT–TTF**, (b) **HMTSF**, and (c) **PT**, and (d) **TSF**. [Reproduced with permission from [27] *ACSNano* **2008**, *2*, 143–155. ©2008, American Chemical Society].

**Figure 32:**
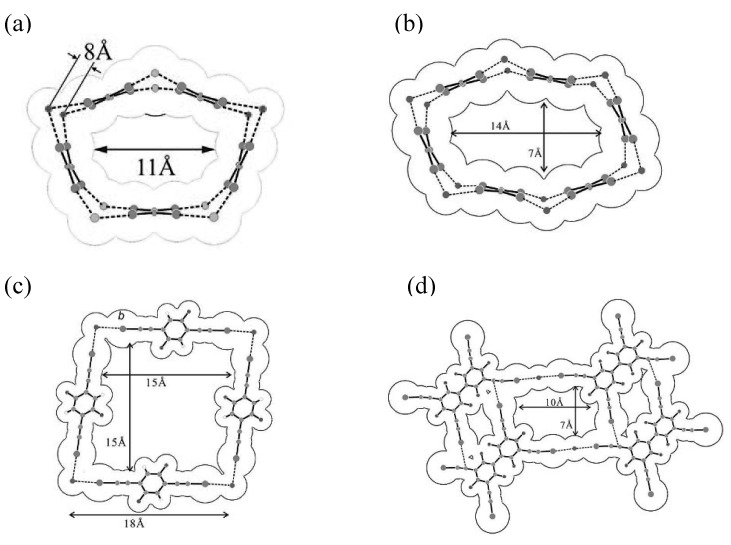
The structures of supramolecular assemblies with van der Waals outlines. The assemblies show (a) pentagonal, (b) hexagonal, and (c and d) parallelogram-shaped channels in crystals **1**, **2**, **3**, and **4**, respectively. [Reproduced with permission from [27] *ACSNano* **2008**, *2*, 143–155. ©2008, American Chemical Society].

Halide anions are well known to form anionic supramolecular assemblies having an infinite repeating structure by coordination with iodine-containing neutral molecules [24]. When halide anions are counteranions of conducting cation radical salts of donor molecules, stacking structures of donor molecules are influenced by the shape of the anionic supramolecular assemblies. By choosing halide anions and iodine-containing neutral molecules in good combination, donor molecules in conducting cation radical salts are one-dimensionally stacked and molecular conductor nanowires are formed. Such examples are found in (**EDT–TTF**)_4_•BrI_2_•(**TIE**)_5_ (**1**), (**HMTSF**)_2_•Cl_2_•(**TIE**)_3_ (**2**), (**PT**)_2_•Cl•(**DFBIB**)_2_ (**3**) and **TSF**•Cl•**HFTIEB** (**4**), where **EDT–TTF**, **HMTSF**, **PT** and **TSF** are donor molecules, while **TIE**, **DFBIB** and **HFTIEB** are iodine-containing neutral molecules (Figure 29) [25,26,27]. Their crystal structures are shown in Figure 30(a)–(d), respectively. In addition, the van der Waals outlines are drawn for **EDT–TTF**, **HMTSF**, **PT** and **TSF** (Figure 31(a)–(d)) and for the supramolecular assemblies of BrI_2_•(**TIE**)_5_ (pentagonal), Cl_2_•(**TIE**)_3_ (hexagonal), Cl•(**DFBIB**)_2_ (parallelogram) and Cl•(**HFTIEB**) (parallelogram) (Figure 32(a)–32(d)). From Figure 31 and Figure 32, it can be easily understood how compatibility is accomplished between the donor molecules and supramolecular assemblies in **1**–**4**.

The resistivity measurements were performed along each axis of the crystals of **1**–**4**, and in all case semiconducting behavior was observed. For **1,** the activation energies (27 meV) were identical for both the directions parallel and perpendicular to the stacking of **EDT–TTF** molecules, but the resistivity (0.1 Ω cm) at room temperature in the parallel direction was 2000-times higher than that in the perpendicular direction. Thus, there is a large anisotropy of 2000 in the resistivity between the two directions. Also for **2** the same activation energies (45 meV) were obtained between the directions parallel and perpendicular to the donor stacking. The anisotropy in the resistivity is about 100 (the room-temperature resistivity in the parallel direction is 1 Ω cm). For **3,** the activation energies were about 300 meV, and the anisotropy was about 10. For **4,** the resistivities at room temperature were 1 × 10^5^Ω cm in the donor stacking direction, and 5 × 10^5^ and 1 × 10^13^Ω cm in the two perpendicular directions, resulting in a resistivity anisotropy of 10^8^. The activation energies were about 300–500 meV in every direction. The high anisotropy observed in these crystals originates from the molecular wire-bundle structures that prevent current flow perpendicular to the stacking direction of the donor molecules. Despite very anisotropic conduction, the activation energies are almost identical in the parallel and perpendicular directions to the donor stacking for **1** and **2**. This coincidence of the same activation energies for the directions parallel and perpendicular to the donor stacking is not insignificant, because the band calculation predicts that the band filling is metallic along the donor stacking direction, while there is no effective transfer integral perpendicular to the donor stacking direction. This phenomenon is already known for K_2_Pt(CN)_4_•Br_0.3_•(H_2_O)_n_ and is considered to be due to lattice defects that limit the conduction along the stacking direction, effectively cutting the nanowire.

#### 2.2.2. Electrochemical Deposition on Gold Wafer Electrode Coated with Porous Alumina Sheet

**Figure 33:**
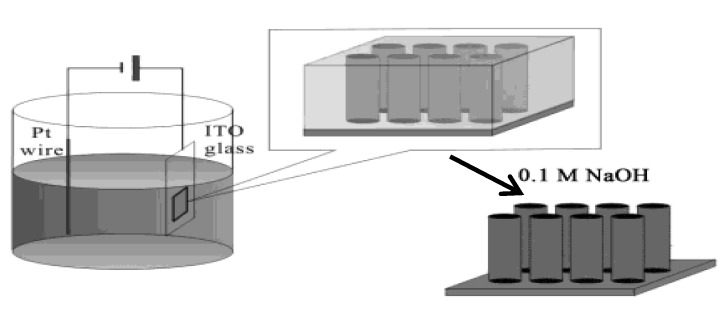
Schematic illustration of the fabricating nanowire arrays of crystalline NMe_4_•[Ni(dmit)_2_]_2_ using a porous alumina template. [Reproduced with permission from [33] *J. Phys. Chem. B* **2004**, *108*, 13638–13642. ©2004, American Chemical Society].

Porous alumina membranes prepared by anodizing Al in the presence of an acidic electrolyte have ordered honeycomb structure characterized by an excellent uniformity in diameter (20 nm to several hundred nm) and spacing of the holes [28,29]. A thin Au or Ag film was sputter-deposited on one side of the porous alumina membrane to serve as an electrode within nano-size reaction space. By electrochemical reactions using Au or Ag electrodes coated with nanoporous alumina membrane, Au and Bi_2_Te_3_ nanowires with a diameter of 40 to 280 ± 30 nm were deposited into the alumina holes [30,31,32]. This method was applied to the preparation of molecule conductor nanowires and nanotubes. A CH_3_CN solution of NMe_4_•[Ni(dmit)_2_] (dmit = 2-thioxo-1,3-dithiole-4,5-dithiolato) in the presence of NMe_4_•ClO_4_ as supporting electrolyte was electrochemically oxidized with a constant current of 5–10 μA cm^–2^ using an Au film coated with nanoporous (diameter = 49 ± 2 nm) alumina membrane as the working electrode and Pt wire (diameter = 1 mm) as the counter electrode (Figure 33) [33].

**Figure 34:**
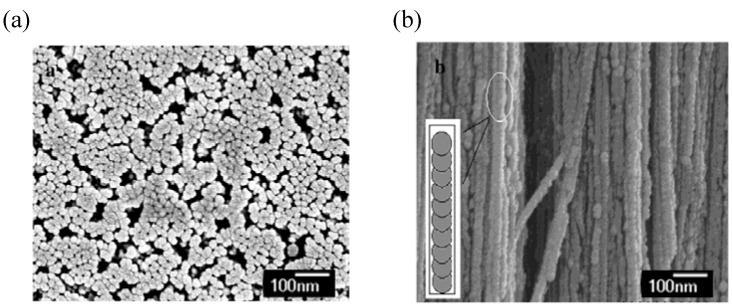
(a) SEM image of the top view of NMe_4_•[Ni(dmit)_2_]_2_ nanowire arrays after the template was partially dissolved. The average pore diameter of template: 49 ± 2 nm. (b) SEM image of the side view of nanowire arrays in the template with the pore diameter of 49 ± 2 nm. [Reproduced with permission from [33] *J. Phys. Chem. B* **2004**, *108*, 13638–13642. ©2004, American Chemical Society].

The [Ni(dmit)_2_]^–^ ion is oxidized to [Ni(dmit)_2_]^δ–^ (0 < δ < –1) and deposited as a salt of NMe_4_•[Ni(dmit)_2_]_2_ into the alumina pores of the anode. Figure 34 shows SEM images of the top and side views of NMe_4_•[Ni(dmit)_2_]_2_ nanowire arrays after the alumina membrane is partially dissolved by the treatment with 0.1 M NaOH aqueous solution. The top view (Figure 34(a)) of the nanowire arrays shows that more than 95% of the pores are filled with the nanowires of NMe_4_^+^ salts, which are densely packed with each other. The tips of the nanowires are almost at the same level and the average diameter of the nanowires is 49 ± 2 nm, which corresponds to the pore diameter of the alumina membrane. The side view (Figure 34(b)) of the nanowires shows that they stand up straight on the Au substrate and separate from each other. The nanowires are about 30 μm long, which also corresponds to the thickness of the alumina membrane, and are continuous.

Looking at the side-view in more detail, the nanowires have periodic corrugated structures with a period of about 30 nm, and look like straight pearl chains. This feature is due to the oscillation of voltage of about 2 mV with a period of about 25 s during the electrochemical deposition under a galvanostatic condition. It is conceivable that such an oscillation of electric current or voltage originates from the following process: the electrochemical deposition of the [Ni(dmit)_2_]^δ–^ ions occurs in a confined one-dimensional environment, so the speed of the diffusion of the electric active species, [Ni(dmit)_2_]^–^ ion is heavily reduced. As the electrochemical deposition is a diffusion-controlled process, the [Ni(dmit)_2_]^–^ ion is rapidly consumed, and the concentration abruptly decreases in the front of the growing surface. The equilibrium electrode potential increases with decreasing the [Ni(dmit)_2_]^–^ ion concentration. At this time the trace impurity of [Ni(dmit)_2_]^2–^ competes with the reaction. During the consumption of [Ni(dmit)_2_]^2–^, the [Ni(dmit)_2_]^–^ ion concentration adjusts itself in the front of the deposition interface and its electrochemical deposition is restarted. This process is repeated until the alumina pores are fully filled.

From the EDS measurement along the axis of a single nanowire, the compositions at different locations through the nanowire are all the same. The observed XRD peaks are indexed to NMe_4_•[Ni(dmit)_2_]_2_ in monoclinic crystal system with the same cell parameters to those of the corresponding single crystal obtained by the electrochemical oxidation with a conventional electrode under the same conditions. These results indicate that the nanowires incorporated into the alumina pores are crystalline NMe_4_•[Ni(dmit)_2_]_2_ salts.

**Figure 35:**
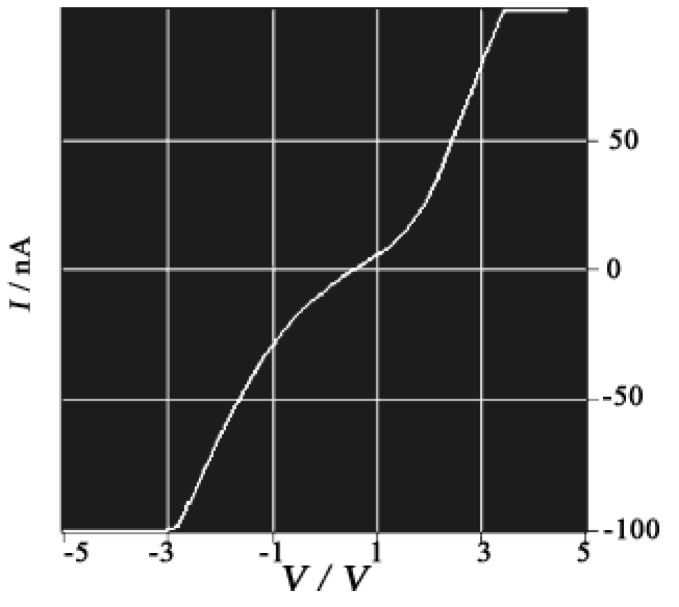
The C–AFM data of the NMe_4_•[Ni(dmit)_2_]_2_ nanowire arrays. After the template was partially dissolved, the C–AFM data was measured on the top of nanowire arrays with the average pore diameter of 49 ± 2 nm. [Reproduced with permission from [33] *J. Phys. Chem. B* **2004**, *108*, 13638–13642. ©2004, American Chemical Society].

The electrical conducting properties of the nanowires were investigated by conductive atomic force microscopy (C–AFM). Figure 35 shows one example of the *I*–*V* curves obtained by the C–AFM data measured on the top of nanowire arrays with the average pore diameter of 49 ± 2 nm. Above ± 3 V the current goes beyond the limit, but in the narrow voltage range of ± (1–3) V the *I*–*V* curve is straight and gives electrical conductivities of 0.1–10 S cm^–1^. This comparatively high electrical conductivity suggests that the long axis of the nanowire corresponds to the conduction direction, that is, the stacking direction of the [Ni(dmit)_2_]^2–^ ions.

The use of Au film electrode coated with the porous alumina membrane was also applied to the preparation of nanotubes of β’’–(**BEDT–TTF**)_4_•[H_2_O•Fe(C_2_O_4_)_3_]•C_6_H_5_NO_2_ [34], whose bulk crystal exhibits metallic conductivity down to low temperature and superconductivity near 7 K [35]. A nitrobenzene solution containing **BEDT–TTF**, (NMe_4_)_3_•[Fe(C_2_O_4_)_3_]•3H_2_O and 18-crown-6-ether was electrochemically oxidized using an alumina/Au film as an anode and a Pt sheet as a cathode with a constant current of 1 μA cm^–1^ for about 72 h. After the reaction completion the alumina membrane was dissolved in 2 M NaOH aqueous solution to get free standing arrays of nanotubes of the **BEDT–TTF** salt on the Au film.

**Figure 36:**
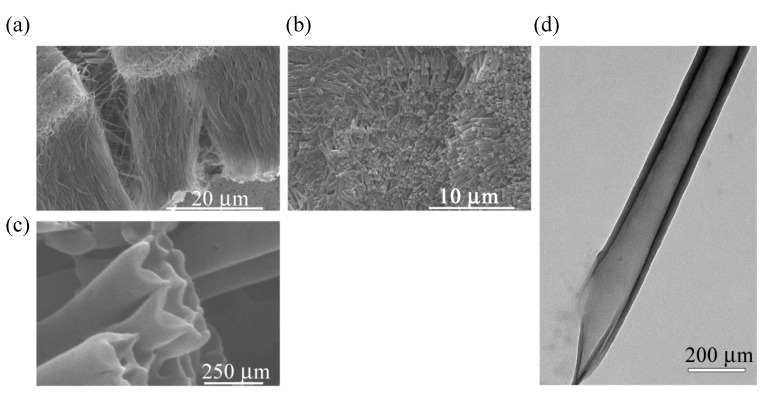
SEM images (a–c), and TEM image (d) of the nanotube arrays. [Reproduced with permission from [34] *Adv. Mater.* **2006**, *18*, 2753–2757. ©2006, Wiley-VCH Verlag GmbH & Co. KGaA].

The morphology of the nanotube arrays are characterized by SEM and TEM. Figure 36(a)–(c) show SEM images of the nanotube arrays prepared by the electrochemical deposition into the alumina pores with a diameter of 200 nm. From the cross-section image in Figure 36(a), the average length of the nanotube is about 30 μm. The top view of the nanotube arrays in Figure 36(b) shows that all of the nanotubes have open ends and a dense arrangement. The details of the ends of the nanotubes are shown in Figure 36(c) and also by the TEM image in Figure 36(d). The outer diameter of the nanotubes is about 200 nm and the thickness is 29 ± 5 nm. No macroscopic defect is observed in all of the nanotubes. The diameter of the nanotubes can be tuned by changing the diameter of the alumina pore, and the length can be controlled by changing the electrochemical oxidation time.

The nanotubes have the same molecular composition to that of the single crystal of β’’–(**BEDT–TTF**)_4_•[H_2_O•Fe(C_2_O_4_)_3_]•C_6_H_5_NO_2_ as shown by the EDX measurement result and from the comparison of Raman spectra and XRD patterns between the nanotube and single crystal. In particular, the presence of mainly (00l) reflections in the XRD pattern indicates that the *c*–axis of the crystal of this **BEDT–TTF** salt is directed perpendicular to the Au film.

**Figure 37:**
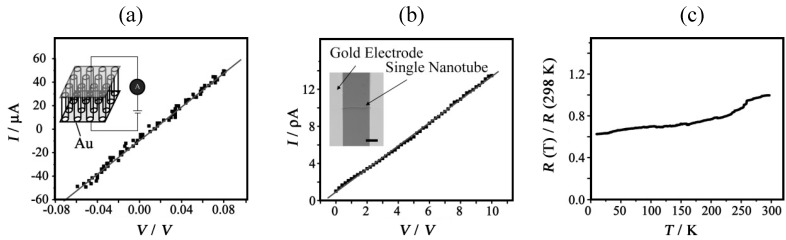
*I–V* characteristics of (a) nanotube arrays and (b) a single nanotube. The inset of (a) shows schematically the circuit used in the measurement and the inset of (b) shows an optical image of the single-nanotube device. The bar length is 10 μm. (c) The temperature dependence of the normalized resistance of nanotube arrays. [Reproduced with permission from [34] *Adv. Mater.* **2006**, *18*, 2753–2757. ©2006, Wiley-VCH Verlag GmbH & Co. KGaA].

The electrical conductivities of the nanotubes were measured. Figure 37(a) and Figure 37(b) show the representative *I*–*V* characteristics of the nanotube arrays and single nanotubes measured at room temperature, respectively. From the slope of the straight line in the *I*–*V* curve in Figure 37(a), the electrical conductivity of the nanotube array is estimated to be about 1.4 × 10^–5^ S cm^–1^. To measure the electrical conductivity of the single nanotube, a device based on an individual nanotube was fabricated on a SiO_2_/Si substrate with a method based on an Au–wire mask (inset in Figure 37(b)). The slope of the straight line in the *I*–*V* curve (Figure 37(b)) gives the electrical conductivity of about 2.9 × 10^–5^ S cm^–1^. The temperature dependence of the normalized resistivity measured along the length direction of the nanotube arrays shows a metallic behavior in the temperature range of 10 K to room temperature (Figure 37(c)).

Almost the same electrical conductivities between the nanotube array and the single nanotube are observed. This implies that the nanotube array is composed of many single nanotubes being connected to both electrodes in parallel, so their electrical conductivities are simply cumulative. However, the electrical conductivity measured by either the nanotube array or the single nanotube is 10^6^-times lower than that of the single crystal. Two main reasons are considered to explain this feature. Firstly, since the length direction of the nanotube is parallel to the *c*–axis, the *I*–*V* characteristics are measured along the *c*–axis, which is the direction of low electrical conductivity (the highly-conducting direction is on the *ab*–plane). Secondly, the crystalline nature of the nanotubes is not as perfect as that of a single crystal, and this may lower the conductivity across the grain boundaries. Furthermore, some effects of the measurement set-up may also be responsible for the decreasing conductivity.

#### 2.2.3. Electrochemical Deposition on Silicon Wafer Electrode Coated with Phospholipid Membrane

**Figure 38:**
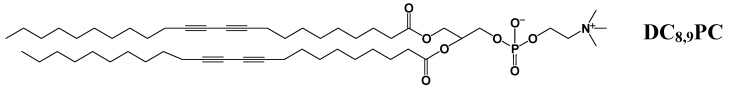
The chemical structure of 1,2-bis(10,12-tricosadiynoyl)-*sn*-glycero-3-phosphocholine (**DC_8,9_PC**).

The use of silicon wafer electrode could occasionally lead to the formation of micro/nano-wires of molecular conductors, as described in 2.1.3. However, a reliable silicon wafer-based method favoring the selective growth of nanowires was necessary. Li and his colleagues modified one-side surface of a silicon wafer with a photochemically-bridged multi-lamellar membrane of phospholipid molecules having two conjugated carbon-carbon triple bonds in each long alkyl chain, 1,2-bis(10,12-tricosadiynoyl)-*sn*-glycero-3-phosphocholine **(DC_8,9_PC**) (Figure 38) and succeeded in the formation of Ni(OH)_2_-based nano-size sublayers incorporated in the open space between the lamellar layers by the electrochemical reduction of an Ni(NO_3_)_2_ aqueous solution using this modified silicon wafer as an electrode [36].

**Figure 39:**
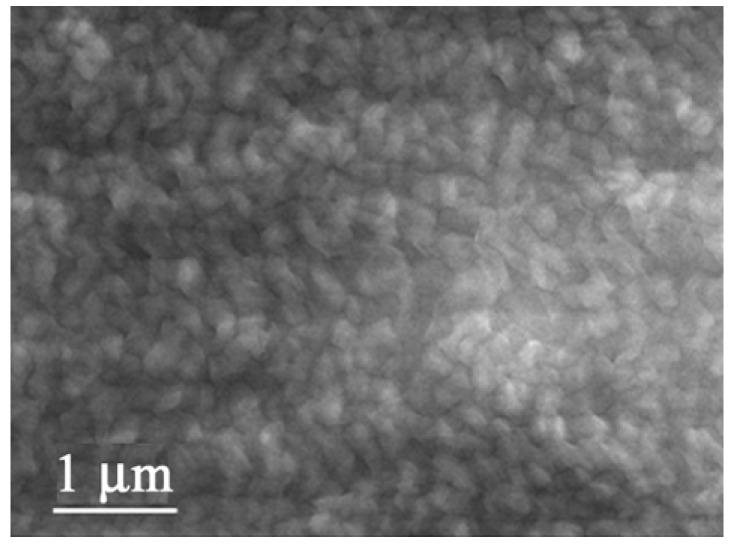
SEM image of **DC_8,9_PC** multilayers coated on a (001)–oriented silicon wafer. [Reproduced with permission from [16] *New J. Chem.* **2007**, *31*, 519–527. ©2007, the Royal Society of Chemistry and the Centre National de la Recherche Scientifique].

**Figure 40:**
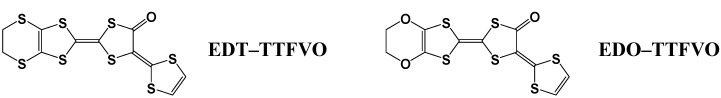
The chemical structure of ethylenedithio- and ethylenedioxy-tetrathiafulvalenoquinone-1,3-dithiolemethides (**EDT–TTFVO** and **EDO–TTFVO**).

As is obvious from the SEM image in Figure 39, this multi-lamellar membrane coated on the silicon wafer possesses nano-size voids surrounded by **DC_8,9_PC** molecules, which can be utilized as the reaction space for an electrochemical oxidation of donor molecules such as to give molecular conductor nanowires selectively. This was actually realized by the electrochemical oxidation of two bent donor molecules, ethylenedithio- and ethylenedioxy-tetrathiafulvalenoquinone-1,3-dithiolemethides (**EDT–TTFVO** [16] and **EDO–TTFVO** [37]) (Figure 40) in the presence of a supporting electrolyte of NBu_4_•FeCl_4_ or NBu_4_•FeBr_4_.

**Figure 41:**
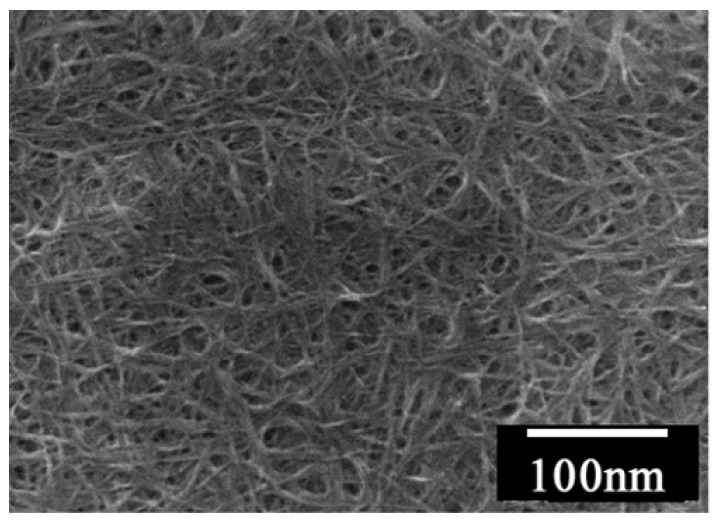
SEM image of (**EDT–TTFVO**)_4_•(FeCl_4_)_2_ nanowires. [Reproduced with permission from [16] *New J. Chem.* **2007**, *31*, 519–527. ©2007, the Royal Society of Chemistry and the Centre National de la Recherche Scientifique].

**Figure 42:**
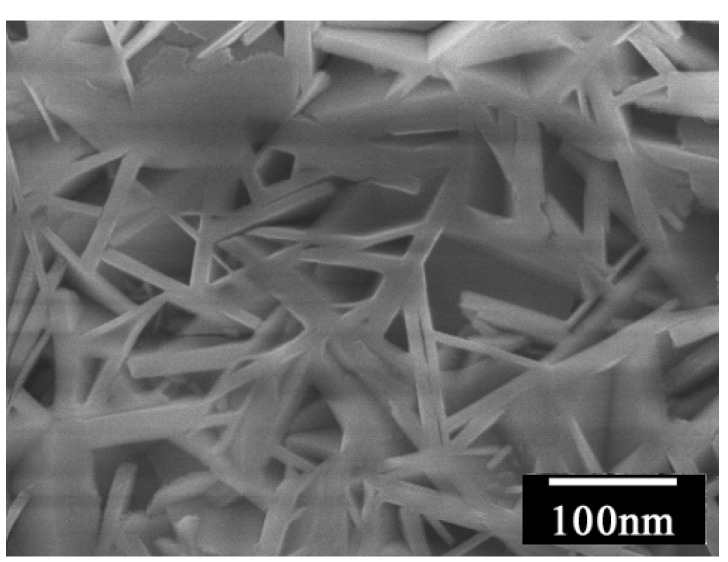
SEM image of (**EDO–TTFVO**)_2_•FeBr_4_•(DCE)_0.5_ nanosticks.

**Figure 43:**
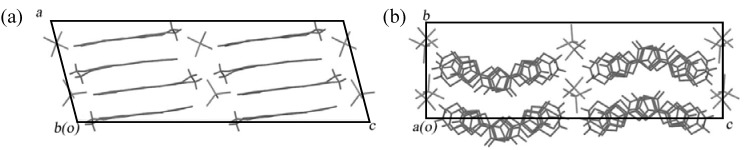
Stacking views of (**EDT–TTFVO**)_4_•(FeCl_4_)_2_ along the (a) *a*− and (b) *b*−axes.

**Figure 44:**
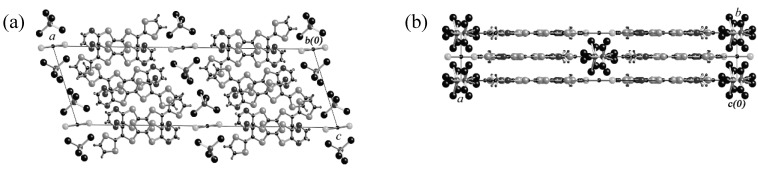
Stacking views of (**EDO–TTFVO**)_2_•FeBr_4_•(DCE)_0.5_ (X = Cl, Br) along the (a) *b*− and (b) *c*−axes.

The electrochemical oxidation of a PhCl–EtOH (9:1, v/v) solution of **EDT–TTFVO** and NBu_4_•FeCl_4_ and of a dichloroethane (DCE) solution of **EDO–TTFVO** and NBu_4_•FeBr_4_ was performed using both electrodes of the as above modified silicon wafer and a native silicon wafer as a reference, respectively. By using the modified silicon wafer electrode nanowires were obtained in the former case and nanosticks in the latter case. Figure 41 and Figure 42 show their SEM images. The diameter of the nanowires is ţ20 nm and that of the nanosticks is about 100 nm. On the other hand, the use of a native silicon wafer gave thin plate-shaped crystals with molecular formulas of (**EDT–TTFVO**)_4_•(FeCl_4_)_2_ and (**EDO–TTFVO**)_2_•FeBr_4_•(DCE)_0.5_, respectively. Their crystal structures are shown in Figure 43 and Figure 44. In (**EDT–TTFVO**)_4_•(FeCl_4_)_2,_ all columns along the *a*–axis are built on the repeated units of one trimer and one monomer, all of which have a head-to-tail stacking mode to each other. Several short S•••S contacts (<3.80 Å of van der Waals contact distance) between donors of neighboring stacks along the *b*–axis, resulting in a two-dimensional network of S•••S interactions. The crystal of (**EDO–TTFVO**)_2_•FeBr_4_•(DCE)_0.5_ contains donor molecules alternating with (FeBr_4_^–^ ion + DCE molecule) layers. The two crystallographically-independent donor molecules form side-by-side arrays along the *c*–axis with several short S•••S(O) contacts, suggesting a strong intermolecular interaction along this direction. In the *bc*–plane the donor molecules construct two identical diagonal stacks along the [0 2 1] and [0 2 –1] directions. This donor array resembles a *β*’’–type packing motif. The calculated Fermi surface is two-dimensional and has a closed-ellipse.

**Figure 45:**
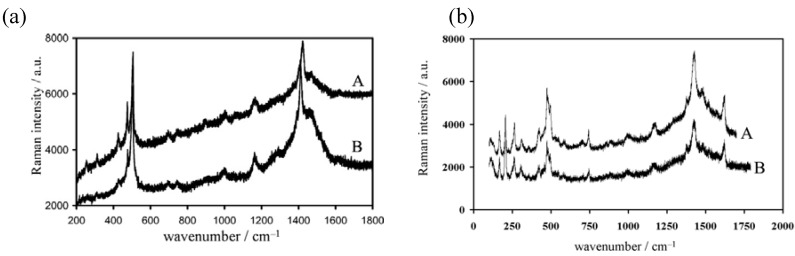
Raman spectra of nanowires or nanosticks (A) and single crystals (B) for (a) (**EDT–TTFVO**)_4_•(FeCl_4_)_2_ and (b) (**EDO–TTFVO**)_2_•FeBr_4_•(DCE)_0.5_.

The nanowires and nanosticks show the same Raman spectra to those of the corresponding single crystals (Figure 45(a) and (b)), so their molecular formulas also apply to the nano-size materials. The growth of the nano-size materials is considered to occur by the following process: the donor molecules migrate from the solution/membrane interface to the silicon electrode surface via the nano-size channels delimited by the long alkyl chains of **DC_8,9_PC** molecules. They are oxidized on the silicon electrode surface to produce the conducting salts by combination with FeCl_4_^–^ or FeBr_4_^–^ ions, being largely present in the vicinity of the hydrophilic silicon surface. As the growing salt is in contact with FeCl_4_^–^ or FeBr_4_^–^ ions located in inter-headgroup areas, the growth can continue but should adapt to nano-size channels. Even when the growing salt reaches the membrane surface, the growth still continues towards the formation of long nanowires lying parallel to the membrane surface, as evidenced by SEM images.

As expected from the stacking structures above, the room-temperature conductivity of (**EDT–TTFVO**)_4_•(FeCl_4_)_2_ is very low (10^–3^–10^–1^ S cm^–1^) and the conducting behavior is semiconducting (activation energy is about 35 meV). On the other hand, (**EDO–TTFVO**)_2_•FeBr_4_•(DCE)_0.5_ shows high room-temperature conductivity (1.8 S cm^–1^) and metallic behavior down to 4 K. The conducting properties of the nano-size materials, in particular, (**EDO–TTFVO**)_2_•FeBr_4_•(DCE)_0.5_ nanosticks, which are expected to be also metallic down to low temperature, are of much interest. The conductivity measurement was done by contacting two probes with the nano-size materials sticking out of the membrane surface, but it was not successful because of resistivity drift. To know the intrinsic conducting properties of a single nanowire or nanostick, it is necessary to fabricate the following device: the part stuck out of the membrane is removed by an appropriate method, and the fattened surface is covered with a gold film. The resistivity of nanowires or nanosticks incorporated into the membrane between the silicon and gold electrodes should be measured, which corresponds to the resistivity of a single nanowire or nanostick separated from each other by insulating phospholipid molecules.

## 3. Different Conducting Properties between Micro-/Nanowires and the Corresponding Single Crystals of Molecular (Magnetic) Conductors and the Plausible Causes

As mentioned for several micro/nano-wires of molecular (magnetic) conductors, the micro/nano-wires and the corresponding single crystals exhibited different conducting properties. In almost all cases, a semiconducting character in the single crystals is also kept in the micro/nano-wires, the room-temperature conductivities and activation energies however becoming very lower and markedly increased, respectively. On the other hand, a metallic character in the single crystals is changed to be semiconducting in the micro/nano-wires. In some cases the reverse case is seen for (**DMe–DCNQI–*d*_7_**)_2_•Cu, where a metallic character is kept down to low temperature for the micro/nano-wires in contrast to the occurrence of M–I transition for the single crystals.

In view of these results the following causes are conceivable for the different conducting properties between micro/nano-wires and the corresponding single crystals of molecular (magnetic) conductors, that is, “the size effect on the conductivity.” In general, molecular (magnetic) conductors based on CT salts possess large anisotropy on the conductivity compared to that of inorganic conductors, because the component donors and acceptors are planar molecules and stack along one- or two-dimensional directions to grow up the crystals of CT salts. The high conductivity is obtained along these directions. The long direction of micro/nano-wires usually corresponds to the stacking direction of donor or acceptor molecules, so the highest conductivity is observed along this direction. The single crystals contain more defects in a larger size than in a smaller size. Since the micro/nano-wires are single crystals as small as possible, the number of defects is supposed to be smallest, giving an ideal molecular conducting wire. However, in the micro/nano-wires a fair part of the mass is used at the surface, whose volume becomes comparable to that of the bulk phase with decreasing the size. It happens that conducting electrons or holes flow much more through the surface than inside the bulk phase. In that case the defect problem will not become so important. Thermal contraction has also a great influence on the electron or hole conduction in micro/nano-wires, which offer a large area in the contact with the electrode. In addition, the different work functions between the molecular conductor and the electrode result in partial doping of electrons or holes into the molecular conductor, and the doping influence is extremely large in micro/nano-wires with small mass. It is still not understood sufficiently which of these causes participates in the` difference in conducting properties between the micro/nano-wires and the corresponding single crystal of each molecular (magnetic) conductor. Further investigation is necessary to clear the causes in more details.

## 4. Conclusion and Prospect

We have presented a review of new development in the preparation of micro/nano-wires of molecular (magnetic) conductors based on CT salts. During this decade a variety of methods using a template or not have emerged to efficiently give molecular micro/nano-wires, which are isolated separately or deposited on a silicon or gold wafer substrate. Nevertheless, such a facile and reliable method is still not available that any CT salt can be obtained as micro/nano-wires. In addition, there are only a few molecular micro/nano-wires exhibiting metallic or narrow bandgap-semiconducting properties. In relation to the conducting property it remains to be not thoroughly understood about size effect due to minutiazation of a bulk crystal. Deeper understanding may become possible by investigating in combination of an acquisition of a good quality of a single nanowire and the corresponding bulk crystal, and detailed conductivity measurements with a theoretical calculation analysis. When these molecular nanowires are intended to be applied to the fabrication of a nano-size transistor, it is very useful to make the molecular nanowires load parallel or perpendicular to a silicon wafer substrate. About 10^4^ pieces of the molecular nanowire-based transistors can be loaded on a silicon wafer substrate with an area of 1 × 1 cm^2^ in the parallel arrangement, and the number of the pieces loaded increases by around 10^3^–times in the perpendicular arrangement, leading to development of a molecule-based high-speed and large information-processing computer. To realized this next generation of computer a facile and reliable method must also be developed to make molecular nanowires load densely and regularly on a silicon wafer substrate. Without a doubt, this field remains a lot of objects to be solved and awaits many researchers to be participated.
